# Covalent conjugation of extracellular vesicles with peptides and nanobodies for targeted therapeutic delivery

**DOI:** 10.1002/jev2.12057

**Published:** 2021-02-16

**Authors:** Tin Chanh Pham, Migara Kavishka Jayasinghe, Thach Tuan Pham, Yuqi Yang, Likun Wei, Waqas Muhammad Usman, Huan Chen, Marco Pirisinu, Jinhua Gong, Seongkyeol Kim, Boya Peng, Weixi Wang, Charlene Chan, Victor Ma, Nhung T.H. Nguyen, Dennis Kappei, Xuan‐Hung Nguyen, William C. Cho, Jiahai Shi, Minh T.N. Le

**Affiliations:** ^1^ Department of Pharmacology Yong Loo Lin School of Medicine National University of Singapore Singapore; ^2^ Department of Biomedical Sciences College of Veterinary Medicine and Life Sciences City University of Hong Kong Hong Kong; ^3^ Institute for Digital Medicine Immunology Programme and Cancer Programme Yong Loo Lin School of Medicine National University of Singapore Singapore; ^4^ N.1 Institute for Health National University of Singapore Singapore; ^5^ City University of Hong Kong Shenzhen Institute Shenzhen China; ^6^ Cancer Science Institute of Singapore National University of Singapore Singapore; ^7^ Department of Clinical Oncology Queen Elizabeth Hospital Hong Kong; ^8^ Vinmec Institute of Applied Science and Regenerative Medicine and College of Health Sciences Vinmec Healthcare system Vin University Hanoi Vietnam; ^9^ Department of Biochemistry Yong Loo Lin School of Medicine National University of Singapore Singapore

**Keywords:** conjugation, delivery, extracellular vesicles, targeted, therapeutics

## Abstract

Natural extracellular vesicles (EVs) are ideal drug carriers due to their remarkable biocompatibility. Their delivery specificity can be achieved by the conjugation of targeting ligands. However, existing methods to engineer target‐specific EVs are tedious or inefficient, having to compromise between harsh chemical treatments and transient interactions. Here, we describe a novel method for the covalent conjugation of EVs with high copy numbers of targeting moieties using protein ligases. Conjugation of EVs with either an epidermal growth factor receptor (EGFR)‐targeting peptide or anti‐EGFR nanobody facilitates their accumulation in EGFR‐positive cancer cells, both *in vitro* and *in vivo*. Systemic delivery of paclitaxel by EGFR‐targeting EVs at a low dose significantly increases drug efficacy in a xenografted mouse model of EGFR‐positive lung cancer. The method is also applicable to the conjugation of EVs with peptides and nanobodies targeting other receptors, such as HER2 and SIRP alpha, and the conjugated EVs can deliver RNA in addition to small molecules, supporting the versatile application of EVs in cancer therapies. This simple, yet efficient and versatile method for the stable surface modification of EVs bypasses the need for genetic and chemical modifications, thus facilitating safe and specific delivery of therapeutic payloads to target cells.

## 1 INTRODUCTION

Extracellular vesicles (EVs), a natural means of intercellular communication and RNA exchange in eukaryotes, are emerging as novel drug delivery vehicles (Andaloussi et al., 2013; Pitt et al., 2016). The natural functions of EVs in mammalian cells suggest their ability to bypass cellular barriers as well as other hurdles to drug delivery, including cytotoxicity, RNase susceptibility, endosomal accumulation, multidrug resistance and immunogenicity (Andaloussi et al., 2013; Pitt et al., 2016). In a recent study, we identified human red blood cells (RBCs) as an ideal source of EVs with promising properties for RNA drug delivery (Usman et al., 2018). We are able to produce RBCEVs on a large scale without the need for cell culture, thereby reducing the cost of production and the risk of contamination. Using RBCEVs for antisense oligonucleotide (ASO) delivery, we observed efficient knockdown of an oncogenic microRNA and suppression of leukaemia and breast cancer development both *in vitro* and *in vivo* (Usman et al., 2018). RBCEVs promise to be a simple and efficient platform for drug delivery that is safe and easily scalable. However, the nonspecific uptake of RBCEVs may cause unwanted side effects in normal tissues when RBCEVs are used to deliver drugs.

To convert EVs into therapies that home specifically onto their target cells, EVs are usually equipped with targeting peptides or antibodies by overexpressing these molecules in the EV‐donor cells using transfection as well as retroviral or lentiviral infection (Andaloussi et al., 2013; Pitt et al., 2016). The most common strategy is to express the targeting epitope with a transmembrane domain or to fuse it with an EV membrane protein such as Lamb2b and CD63, so that the epitope is displayed on the surface of EVs. A well‐known example is the successful generation of brain‐targeting EVs from dendritic cells transfected with a plasmid encoding EV protein Lamb2b and brain‐targeting RVG peptide. The engineered EVs can cross the blood‐brain barrier and deliver RNA therapeutics into the brain (Alvarez‐Erviti et al., 2011). This study initiated an exciting wave of research on brain‐targeting EVs that was thought to facilitate the development of new therapies against neurological diseases (Cooper et al., 2014). A similar approach was used to generate tumour‐targeting EVs. For example, a fusion of the platelet derived growth factor receptor transmembrane domain and an epidermal growth factor receptor (EGFR)‐targeting peptide is expressed from a retroviral plasmid in HEK‐293T cells to produce EGFR‐targeting EVs that deliver anti‐cancer let‐7 to breast tumours (Ohno et al., 2013). Similarly, a Neuro2A (N2A) cell line expressing membrane‐anchored‐α‐EGFR nanobody also produces EVs with a high affinity for EGFR on cancer cells (Kooijmans et al., 2016).

Genetic engineering of EV‐donor cells in the pioneering studies above is tedious and costly with multiple steps of cloning, transfection or viral transduction, selection, large‐scale cell culture and EV purification. Although it can enable stable conjugation of EVs with targeting moieties, genetic manipulation poses a high risk of horizontal gene transfer because the EVs may incorporate high‐copy plasmids or transgenes that are eventually transferred to target cells. If EVs are produced by immortalized cell lines, their oncogenic factors including mutated DNA, RNA, and proteins could be packed into EVs and delivered into target cells, leading to the risk of tumorigenesis (Balaj et al., 2011). Moreover, most stem cells and primary cells are not amenable to genetic engineering methods, as they are typically difficult to transduce (Kooijmans et al., 2018).

An alternative approach is to engineer EVs post‐isolation via chemical or affinity‐based methods. There are several methods of coating EVs with EV‐binding peptides or antibodies based on affinity binding, but these conjugations are transient and unstable (Antes et al., 2018; Chiu et al., 2002; Gao et al., 2018; Kooijmans et al., 2018; Yamamoto et al., 2016; Yerneni et al., 2019; Zou et al., 2019). For example, Kooijman et al. developed an α‐EGFR nanobody fused to the C1C2 domain of lactadherin that binds to phosphatidylserine (PS) on the surface of EVs (Kooijmans et al., 2018). The α‐EGFR‐C1C2‐EVs were applied to target EGFR‐positive cancer cells*in vitro* but not tested *in vivo*. It is unclear if such an EV‐nanobody complex could survive in the circulation and accumulate in the tumour. Moreover, nanobodies with a hydrophobic domain such as C1C2 often form aggregates and the efficiency of affinity‐based conjugation varies depending on the abundance of the binding ligand, such as PS in this case. Chemical engineering methods have been developed to covalently conjugate peptides to EVs (Nakase et al., 2016; Nakase et al., 2016; Smyth et al., 2014). However, they are not widely used due to the excessive harshness of chemical treatments to the surface of EVs, which could cause undesirable loss of function (Armstrong et al., 2017). Therefore, a method for stable and gentle conjugation of EVs is highly desirable.

Here, we have developed a **simple enzymatic method** to conjugate peptides and nanobodies onto EVs without any genetic or chemical modification of donor cells. We used protein ligating enzymes, including Sortase A and OaAEP1 ligase, to create permanent covalent bonds between EVs and peptides. The catalytic reaction occurred at a neutral pH, avoiding any risk of EV damage. OaAEP1 ligase was particularly efficient in catalyzing the reactions, ligating ∼380 copies of peptides to each EV. We used this method to conjugate RBCEVs with an EGFR‐targeting peptide to facilitate the specific uptake of RBCEVs by EGFR‐positive cells. We also conjugated RBCEVs with a self‐peptide that prevented phagocytosis and increased the availability of RBCEVs in the circulation. We further developed a 2‐step‐ligation protocol to conjugate RBCEVs with α‐EGFR, α‐mCherry, and α‐HER2 nanobodies that facilitates the specific uptake of EVs by target cells expressing the corresponding receptors. Surface‐modified RBCEVs could be loaded with mRNA or paclitaxel (PTX), promoting the specific delivery of these payloads to target cells. Furthermore, we demonstrated that EGFR‐targeting peptides and nanobodies facilitated specific uptake of RBCEVs by EGFR‐positive lung cancer cells *in vivo*. Targeted delivery of EV‐encapsulated PTX at a low dose (10‐20 times lower than the clinical‐equivalent dose) significantly enhanced the drug efficacy in suppressing tumour growth in an EGFR‐positive lung cancer xenografted mouse model. Thus, the conjugation of EVs with targeting peptides or nanobodies is useful for achieving targeted drug delivery with high specificity. The enzymatic EV modification method we described here is simple and gentle but efficient for conjugation of EVs with either peptides or nanobodies. The stable covalent conjugation facilitates the accumulation of functional peptide/nanobody‐coated EVs at target sites, leading to their specific uptake by target cells and subsequent delivery of therapeutic molecules. Importantly, this conjugation method does not cause any EV damage, toxicity or any risk of oncogenesis.

## 2 RESULTS

### 2.1 Protein ligases and RBCEVs are produced at high purity

**FIGURE 1:**
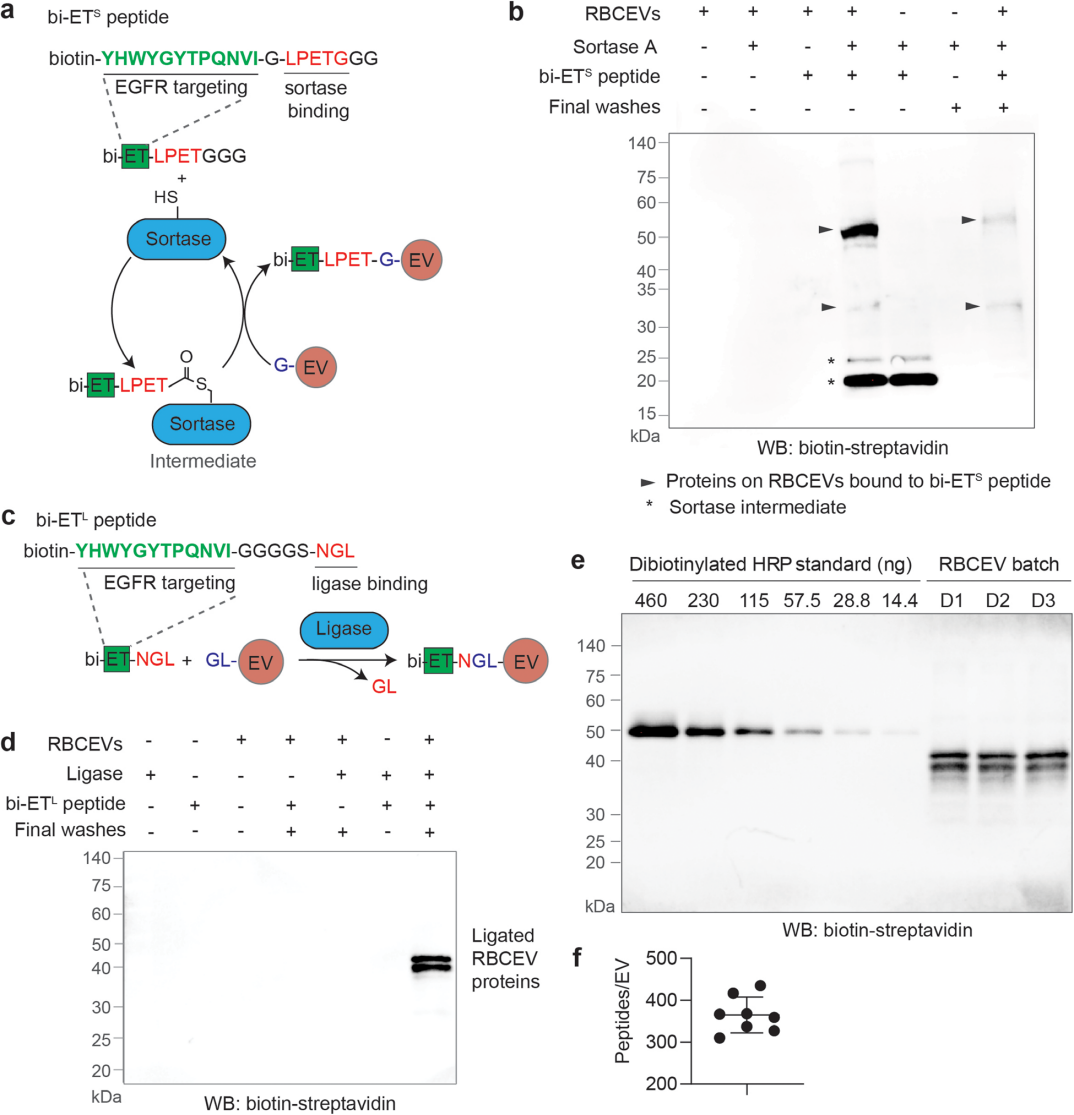
Protein ligating enzymes mediate a covalent conjugation of RBCEVs with peptides. **(a)** Design of an EGFR‐targeting (ET) peptide with a sortase binding site and biotin (bi) conjugation (bi‐ET^S^ peptide). Sortagging reaction occurs between the bi‐ET^S^ peptide and proteins with N‐terminal Glycine (G) on RBCEVs, mediated by Sortase A. **(b)** Western blot (WB) analysis of biotin following an SDS‐PAGE separation of RBCEV proteins conjugated with the bi‐ET^S^ peptide. Sortase intermediates were removed in three washes with PBS. Biotin was detected using HRP‐conjugated streptavidin. Molecular weights (kDa) of protein markers are shown on the left. **(c)** Design of a typical OaAEP1‐ligase‐mediated reaction between a biotinylated ET peptide with a ligase binding site (bi‐ET^L^ peptide) and proteins containing N‐terminal GL (preferred but not required) on RBCEVs. **(d)** Western blot analysis of biotin resulted from the OaAEP1‐ ligase‐mediated conjugation of RBCEVs with the bi‐ET^L^ peptide, similar to (b). **(e)** Western Blot analysis of RBCEVs from three different donors (D1‐D3) ligated with a biotinylated control peptide using OaAEP1 ligase. Dibiotinylated HRP was used as a reference for quantification, and a particle analyzer was used to obtain the number of ligated EVs loaded per well. **(f)** Average number of peptides ligated to each EV ± SEM (n = 8 donors).

For RBCEV conjugation, we employed two protein‐ligating enzymes, Sortase A heptamutant and OaAEP1 Cys247Ala ligase that are known to catalyze covalent‐bonding reactions between two peptides, one bearing the enzyme‐recognition motif on its C terminus and another one with a free glycine‐bearing N terminus (Figure 1a,c) (Popp et al., 2007; Yang et al., 2017). We produced Sortase A and OaAEP1 ligase from *E. coli* at high purity and high yield using affinity and size exclusion chromatography (Figure S1A‐C). Since Sortase A has been used to conjugate mature RBCs with peptides (Pishesha et al., 2017), we hypothesized that the RBCEV surface might possess RBC membrane proteins that can act as substrates for Sortase A or OaAEP1 ligase. RBCEVs purified according to our previous protocol (Usman et al., 2018) were intact and free of debris with a typical cup‐shaped appearance under a transmission electron microscope, TEM (Figure S2A). RBCEVs express glycophorin A (GPA) on their surface as a unique marker of human RBCs (Figure S2A). We have shown before that RBCEVs also expressed some common markers of EVs including ALIX and TSG101, but not the endoplasmic reticulum marker CANX (Usman et al., 2018). They were relatively homogenous in size, 120–200 nm in diameter, as we observed using TEM and a Nanosight particle analyzer (Figure S2A‐B). Using label‐free quantitative (LFQ) mass spectrometry (Cox et al, 2014), we identified nearly 100 proteins that were highly abundant in RBCEV protein extracts from 4 separate donors, including some well‐known RBC proteins (Figure S2C, Table S2).

### 2.2 Sortase A and OaAEP1 ligase mediate the conjugation of EVs with peptides

We designed biotinylated peptides containing a known EGFR targeting (ET) site and a recognition motif for Sortase A or OaAEP1 ligase at the C terminus (Figure 1a, c and Table S1) (Li et al., 2005; Popp et al., 2007). Of note, OaAEP1 ligase also binds to Sortase's recognition motif (Hemu et al., 2019). In a Western blot analysis using HRP‐conjugated streptavidin, we observed multiple biotinylated protein bands after the sortagging or ligation reaction (Figure 1b, d). Sortagging reactions created 2 bands at 20–25 kDa as intermediate products, and 2 more bands at ∼35 kDa and ∼50 kDa remained after washing, indicating RBCEV surface proteins conjugated with the biotinylated ET peptide (Figure 1b). In the reactions catalyzed by OaAEP1, after optimization, we observed only 2 intense protein bands at ∼35 and ∼45 kDa, both of which remained after extensive washing (Figure 1d & S3A). These bands are slightly different from the sortagging products, likely because the OaAEP1 ligase preferably acts on proteins with both N‐terminal glycine and leucine residues (GL) and OaAEP1 reactions do not generate any stable intermediate (Yang et al., 2017). Both the sortagged and ligated products survived under the denaturing condition of SDS‐PAGE, indicating the formation of stable covalent bonds between RBCEV membrane proteins and ET peptides. Subsequently, we ligated the ET peptide to 3 different batches of RBCEVs purified from three independent donors and found the same spectrum of ligated protein bands, suggesting that RBCEVs from each donor contained the same surface proteins that consistently reacted with biotinylated ET peptides after incubation with the OaAEP1 ligase (Figure 1e).

To quantify the number of ET peptides that were conjugated to RBCEVs using OaAEP1 ligase, we compared the biotin signals from the ET‐RBCEVs to a serial dilution of dibiotinylated HRP. This comparison indicated that there were ∼380 copies of peptides ligated to each RBCEV on average (Figure 1e‐f). Similarly, we quantified the conjugation efficiency of a biotinylated peptide to RBCEVs using Sortase A and compared the sortagged products with a serial dilution of dibiotinylated HRP (Figure S2D). The average sortagged peptide number was estimated to be ∼65 copies per EV, much less than the peptide number resulted from OaAEP1‐ligase‐mediated conjugation.

**FIGURE 2:**
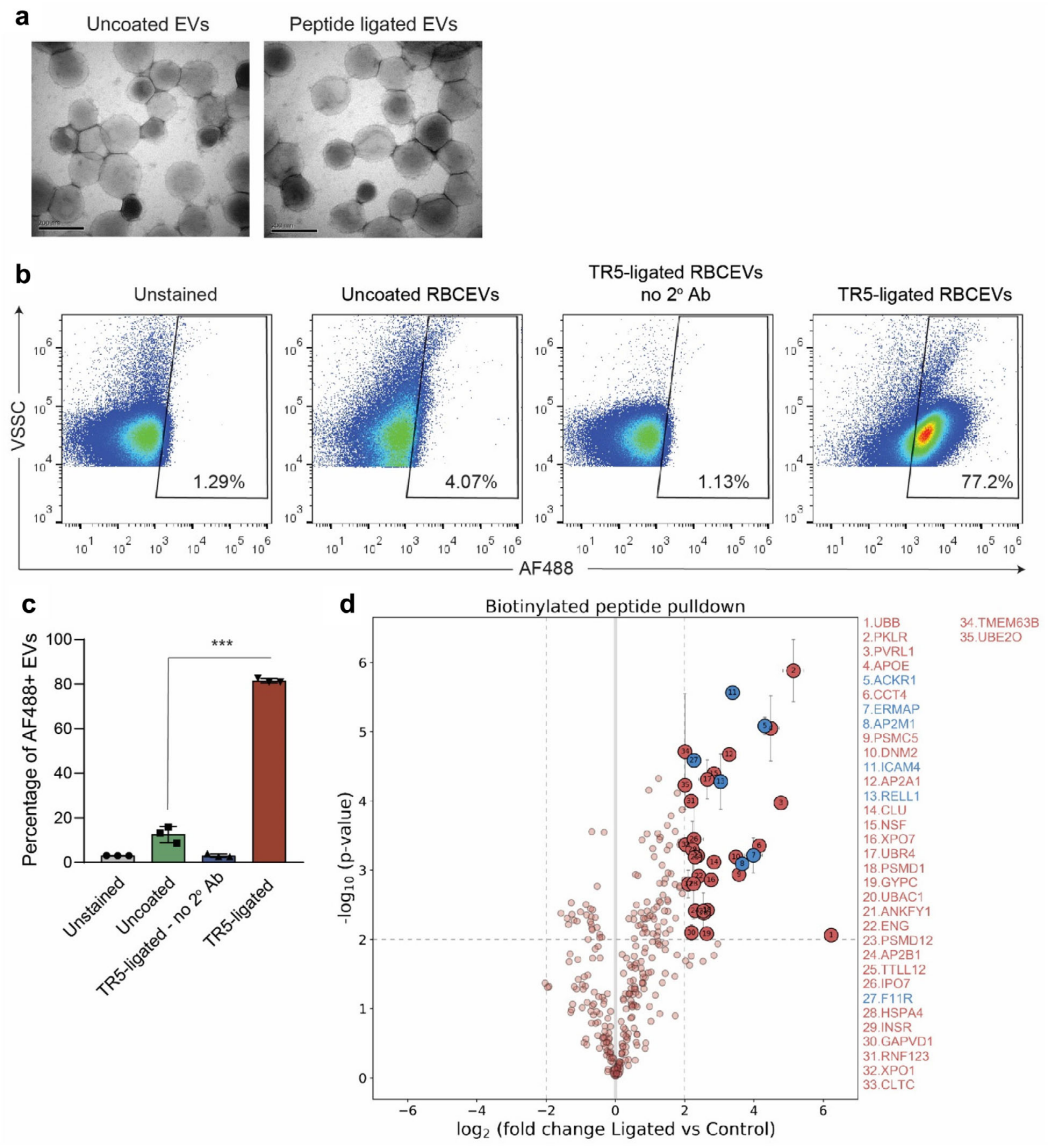
Characterization of peptide‐conjugated RBCEVs. **(a)** Representative TEM images of uncoated and coated RBCEVs (conjugated with biotinylated peptide TR5 using OaAEP1 ligase). Scale bar, 200 nm. **(b‐c)** Single‐EV FACS analysis of RBCEVs conjugated with biotinylated peptide (using OaAEP1 ligase). The biotin on RBCEVs was detected via sequential incubation of the EVs with streptavidin followed by a biotinylated antibody which was subsequently detected using an AF488‐conjugated secondary antibody (2^o^ Ab). Background noise (VSSC < 10^4^) was excluded to obtain a distinct EV population.**(d)** Identification of RBCEV proteins associated with biotinylated TR5 peptide after OaAEP1‐mediated ligation using biotin‐streptavidin pulldown assay and label‐free quantitative mass spectrometry analysis. Volcano plot of biotin pulldown with lysates from TR5‐ligated RBCEVs (ligated) compared to uncoated RBCEVs incubated with biotinylated TR5 peptide without ligase addition (control). Specifically enriched proteins (numbered circles) are distinguished from background binders by a two‐dimensional cut‐off of > 4‐fold enrichment and *P* < 0.01. Two‐dimensional error bars represent the standard deviation based on iterative imputation cycles during the label‐free analysis to substitute zero values. Membrane proteins with the molecular weight of 25–50 kDa are highlighted in blue. Student's *t*‐test *** *P* < 0.001

TEM images of peptide‐coated and uncoated RBCEVs showed similar morphology, indicating that the ligation did not affect the EVs’ structure or integrity (Figure 2a). To estimate the efficiency of conjugation, we analyzed peptide‐coated RBCEVs using single‐EV flow cytometry according to the MIFlowCyt‐EV guidelines (Welsh et al., 2020). RBCEVs were clearly distinguished from background noise, and were identified as a distinct population (Figure S3B). We detected the biotinylated‐peptide (TR5) on the surface of the RBCEVs via a sequential incubation with streptavidin and a biotinylated antibody which in turn was detected using an AF488‐ conjugated secondary antibody. The TR5‐ligated EVs showed a significant increase in fluorescent intensity, with ∼80% of RBCEVs positive for AF488 fluorescence (Figure 2b‐c). Uncoated RBCEVs amplified in a similar way did not show an increase in fluorescence; neither did the reagent control which consisted of the antibody only incubated in PBS (Figure S3C).

To identify proteins on RBCEVs that were ligated to the biotinylated peptide, we pulled down the biotinylated peptide‐ligated RBCEV proteins using streptavidin magnetic beads and analyzed precipitated proteins using label‐free quantitative mass spectrometry (Figure 2d, Table S3). Among proteins that were significantly enriched in the biotin‐streptavidin pulldown (fold change > 4, *P*‐value < 0.01), we found 6 membrane proteins within the molecular weight range of 25–50 kDa (ACKR1, AP2M1, ERMAP, F11R, ICAM4 and RELL1), similar to that of the ligated products observed using Western blot. Hence, these proteins are likely substrates of the OaAEP1 ligase on the RBCEV membrane. Of note, an N‐terminal glycine is not absolutely required for the ligase binding (Yang et al., 2017); hence, we did not exclude membrane proteins without N‐terminal glycine from the 6 identified membrane proteins. Other proteins identified in the pulldown may have been enriched due to their association with the ligated proteins, but were not ligated themselves.

To evaluate the applicability of this conjugation method to other types of EVs, we isolated EVs from leukaemia THP1 cells and ligated them to a biotinylated peptide harbouring a ligase‐binding site. This reaction resulted in multiple biotinylated protein bands from 25 to 75 kDa, suggesting that THP1 EVs contain many different membrane proteins that act as substrates for the ligase reaction (Figure S3E‐F).

### 2.3 Conjugation of EVs with EGFR‐targeting peptides promotes their uptake by EGFR‐positive cells

**FIGURE 3:**
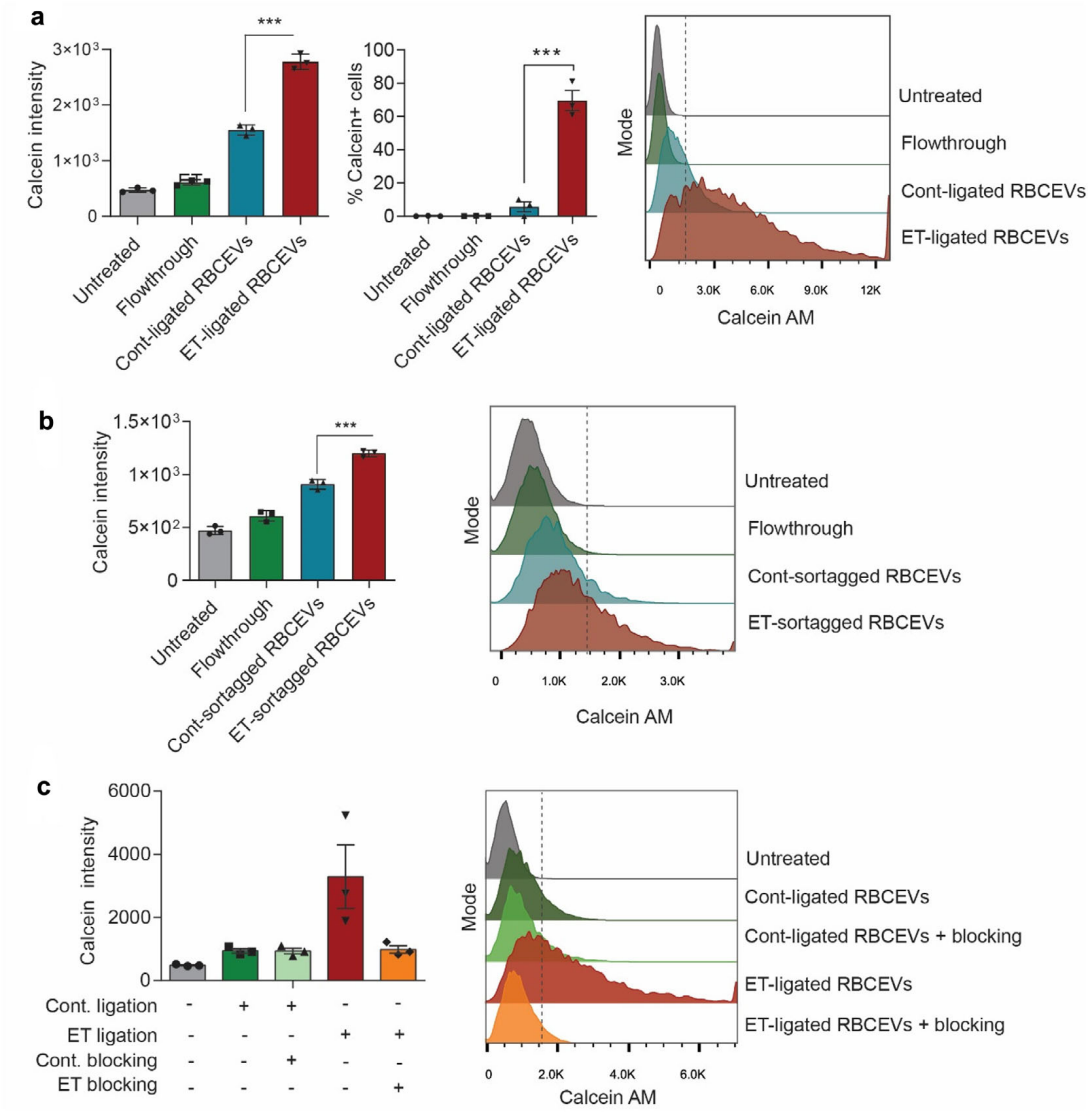
Conjugation of RBCEVs with EGFR‐targeting peptides increases the uptake of the EVs by EGFR‐positive cells. **(a)** Uptake of Cont. or ET‐ligated‐RBCEVs (using OaAEP1 ligase) by EGFR^+^ H358 cells, quantified based on FACS analysis of Calcein AM, the fluorescent label of RBCEVs. **(b)** Uptake of Cont/ET‐sortagged‐RBCEVs (using Sortase A) by H358 cells, quantified using FACS analysis of Calcein AM. **(c)** Effect of blocking peptides, which compete for binding to EGFR, on the uptake of ligated RBCEVs, indicated by Calcein AM intensity in H358 cells treated with Cont/ET‐peptide‐ligated RBCEVs. In each uptake assay, 200,000 cells were incubated with 5 μg Calcein‐AM labelled RBCEVs (2.5 × 10^9^ particles) at 37°C for 2 h. Control cells were treated with the flowthrough of the last wash of Calcein‐AM‐labelled RBCEVs. The graphs present the mean ± SEM (n = 3 donors). Student's one‐tailed *t*‐test *P < 0.05, ***P < 0.001

EGFR is a tyrosine kinase encoded by a proto‐oncogene that is elevated in many types of solid cancers, and its elevation is considered a common cancer biomarker (Normanno et al., 2006). Among cell lines available in our lab, we found that human EGFR was highly abundant in lung cancer H358 and HCC827 cells, but negative in leukaemia MOLM13 and neuroblastoma N2A cells (Figure S4A). Consistently, biotinylated ET peptide bound to the surface of H358 and HCC827 cells but not MOLM13 and N2A cells (Figure S4B). To test the cellular uptake of RBCEVs, we labelled the peptide‐conjugated RBCEVs with Calcein AM, which became fluorescent only in the presence of esterases when it was loaded into RBCEVs or internalized into cells. The fluorescence‐labelled ET‐RBCEVs were washed extensively with size exclusion chromatography (SEC) and centrifugation. Unbound Calcein AM was eluted in fractions 18–20 of the SEC, separated well from RBCEVs which were eluted in fraction 7–9 (Figure S4C). We incubated H358 cells with a suboptimal (non‐saturated) dose of the fluorescent ET‐RBCEVs for 2 h and analyzed fluorescence in H358 cells. The Calcein fluorescence intensity in H358 cells treated with the ET‐peptide‐ligated‐RBCEVs or ET‐peptide‐sortagged‐RBCEVs was significantly higher than that in H358 cells incubated with control‐peptide‐coated RBCEVs (Figure 3a‐b). Furthermore, the percentage of Calcein‐positive cells was also significantly higher in EGFR‐positive H358 cells compared to EGFR‐negative N2A cells treated with ET‐peptide‐ligated RBCEVs and H358 cells incubated with control‐peptide‐ligated RBCEVs (Figure 3a and Fig. S5A). The fold change in ET‐RBCEV uptake was higher with OaAEP1‐mediated ligation than with Sortase A‐mediated conjugation (Figure 3b). Hence, we demonstrated that the conjugation of EVs with an EGFR‐specific peptide did not disrupt the EV's payload delivery function and in fact significantly increased the specific uptake of the EVs by EGFR‐positive cells. To further confirm that the EV uptake specificity resulted from the interaction between EGFR and the ET peptide, and not from other binding interactions due to the formation of de novo peptides after ligation, we added 120 μM free ET peptide to the incubation of H358 cells with ET‐RBCEVs. The competitive binding of the free ET peptide blocked the uptake of fluorescent ET‐RBCEVs by H358 cells (Figure 3c), indicating that the EGFR‐ET binding interaction is necessary for cellular uptake of ET‐RBCEVs.

**FIGURE 4:**
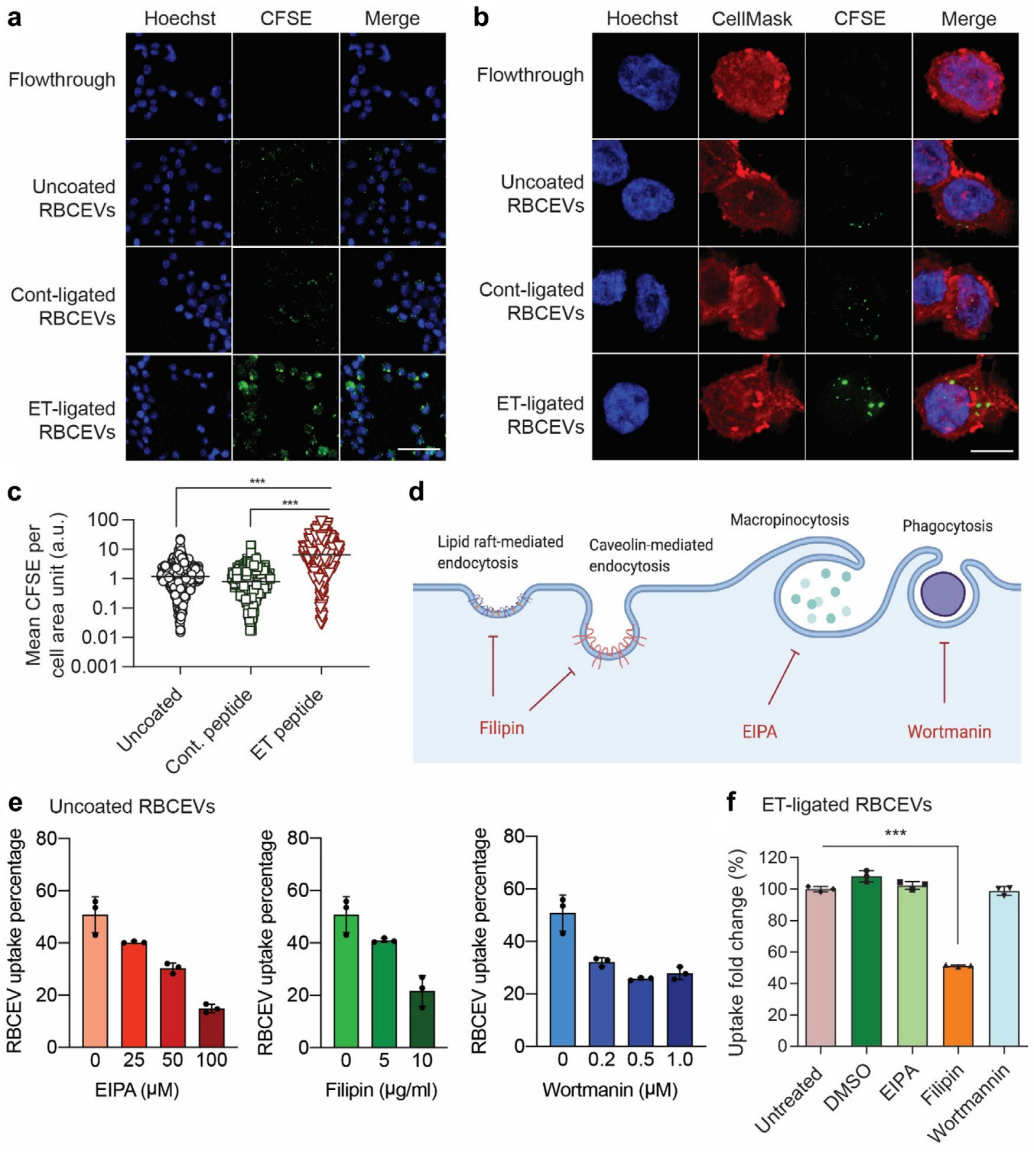
Conjugation of RBCEVs with EGFR‐targeting peptides increases the uptake of the EVs by EGFR‐positive cells.**(a)** Representative images of the uptake of CFSE‐labelled RBCEVs by H358 cells, obtained using confocal microscopy. Scale bar, 50 μm. **(b)** Representative Z‐stacked images of H358 cells taking up CFSE‐labelled RBCEVs, also stained with CellMask (red) and Hoechst (blue). Scale bar, 10 μm. **(c)** Mean CFSE signal per cell area unit was determined from the quantification of CFSE intensity in 1200‐1600 cells per condition. **(d)** Possible effects of chemical inhibitors, EIPA, Filipin and Wortmannin on the uptake of RBCEVs via separate routes of endocytosis. Image was created using biorender.com. **(e)** Uptake of uncoated Calcein‐AM‐labelled RBCEVs by H358 cells after treatments with EIPA, Filipin and Wortmanin at indicated concentrations, determined using flow cytometry and presented as the percentage of Calcein‐AM‐positive cells. **(f)** Uptake of Calcein‐AM‐labelled RBCEVs that are conjugated with ET peptide by H358 cells after treatments with 100 μM EIPA, 10 μg/ml Filipin, and 0.5 μM Wortmanin, presented as fold change in Calcein AM intensity relative to the untreated control. In each uptake assay, 200,000 cells were incubated with 5 μg RBCEVs at 37°C for 2 h with or without 1‐h prior treatment with indicated inhibitors. Peptide conjugation was performed using OaAEP1 ligase. The graphs present the mean ± SEM (n = 3 donors). Student's one‐tailed *t*‐test ***P < 0.001

We also visualized the uptake of RBCEVs using immunofluorescence analysis (Figure 4a‐c). Following an incubation of H358 cells with CFSE‐labelled RBCEVs, CFSE was observed as bright green fluorescence in a punctate pattern, with higher intensity and frequency in ET‐RBCEV treated samples. The accumulation of CFSE signals was observed mainly inside the cells as shown in a video scanning through the Z‐stack of fluorescent images (Movie S1). Indeed, the punctate pattern of CFSE indicates an accumulation of RBCEVs at high density in intracellular vesicles, suggesting that RBCEVs were internalized into the cells via endocytosis.

To identify the route of RBCEV uptake, we added three different endocytosis inhibitors to the incubation of H358 cells with RBCEVs: Filipin, which blocks caveolin‐mediated and lipid raft‐mediated endocytosis; 5‐(N‐ethyl‐N‐isopropyl) amiloride (EIPA), which blocks macropinocytosis and wortmannin, which blocks phagocytosis (Figure 4d). All three inhibitors reduced the uptake of Calcein‐AM‐labelled uncoated RBCEVs in a dose dependent manner (Figure 4e). These effects were not due to any changes in cell viability at the selected doses (Figure S5B‐E). However, only Filipin reduced the uptake of ET‐peptide‐coated‐RBCEVs (Figure 4f). Therefore, the uptake of uncoated RBCEVs depends on multiple endocytic pathway but the uptake of ET‐peptide‐conjugated RBCEVs depends only on caveolin‐mediated and lipid raft‐mediated endocytosis.

### 2.4 Conjugation of EVs with a ‘self’ peptide reduces their phagocytosis and increases EV circulation

**FIGURE 5:**
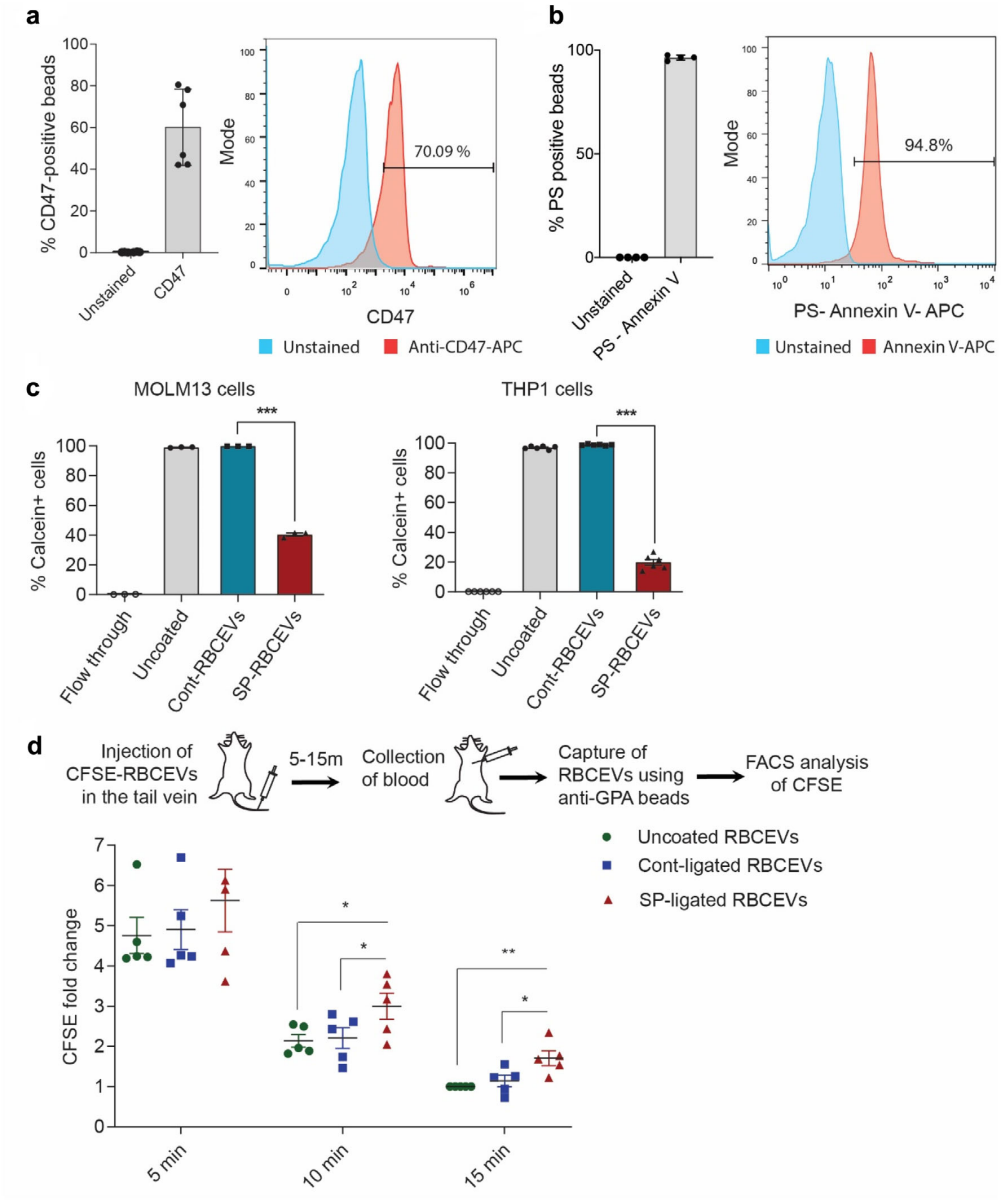
Conjugation with self‐peptide prevents phagocytosis of RBCEVs and enhances the availability of RBCEVs in the circulation. **(a)** Flow cytometry analysis of CD47 on RBCEV‐bound beads. **(b)** Flow cytometry analysis of Annexin V binding to PS on RBCEVs that were immobilized on latex beads. **(c)** FACS analysis of Calcein AM in MOLM13 and THP1 monocytes that were treated with control or self‐peptide (SP) ligated Calcein‐labelled RBCEVs. 200,000 cells were incubated with 5 μg RBCEVs (2.5 × 10^9^ particles) at 37°C for 2 h. The graphs present the average percentage of Calcein‐positive cells ± SEM (n = 3 to 6 donors). **(d)** FACS analysis of CFSE‐labelled RBCEVs that were captured by anti‐GPA‐antibody‐coated streptavidin beads from the plasma of NSG mice, 5–15 min after a tail vein injection of 0.5 mg CFSE‐labelled human RBCEVs (2.5 × 10^11^ particles). RBCEVs were uncoated or ligated with the control or self‐peptide. The graph presents the mean intensity of CFSE ± SEM (n = 5 mice). Student's one‐tailed *t*‐test *P < 0.05, **P < 0.01, ***P < 0.001

We further investigated if RBCEVs displayed CD47 and PS as ‘don't eat me’ and ‘eat me’ signals, respectively. These signals determine the recognition of EVs for clearance by leukocytes (Rodriguez et al., 2013 ). Staining of RBCEVs on latex beads with an anti‐CD47 antibody revealed that ∼60% of the beads were positive for CD47 on average (Figure 5a); whereas, nearly 95% of RBCEV‐bound latex beads were stained with Annexin V which binds to PS (Figure 5b). These data suggest that PS was relatively more abundant than CD47 on the surface of RBCEVs. Hence, we tested if RBCEVs can be conjugated with a ‘self’ peptide derived from CD47, to increase the ‘don't eat me’ signals on RBCEVs and avoid phagocytosis by leukocytes (Rodriguez et al., 2013). Conjugation with the self‐peptide significantly reduced the uptake of RBCEVs by two monocytic cell lines, MOLM13 and THP1 cells (Figure 5c). We further labelled self‐peptide‐coated RBCEVs with CFSE and injected them into the tail vein of immunodeficient NOD‐scid‐gamma (NSG) mice. After 5, 10 and 15 min, we captured RBCEVs in the blood using anti‐human‐GPA‐antibody‐coated magnetic beads (Figure 5d). As GPA is a RBC specific marker that is expressed in RBCEVs but not in other EVs, and the antibody is specific to human GPA, we could separate the injected human RBCEVs from mouse EVs in the blood and quantify them based on FACS analysis of CFSE fluorescence. The analysis revealed that self‐peptide‐ligated RBCEVs were significantly more abundant than control‐peptide‐ligated RBCEVs in the blood of injected mice (Figure 5d). These data indicate that the self‐peptide can be used to reduce the non‐specific phagocytosis of EVs and increase the circulatory flux of RBCEVs.

### 2.5 Conjugation of nanobodies to EVs using a two‐step ligation method

**FIGURE 6:**
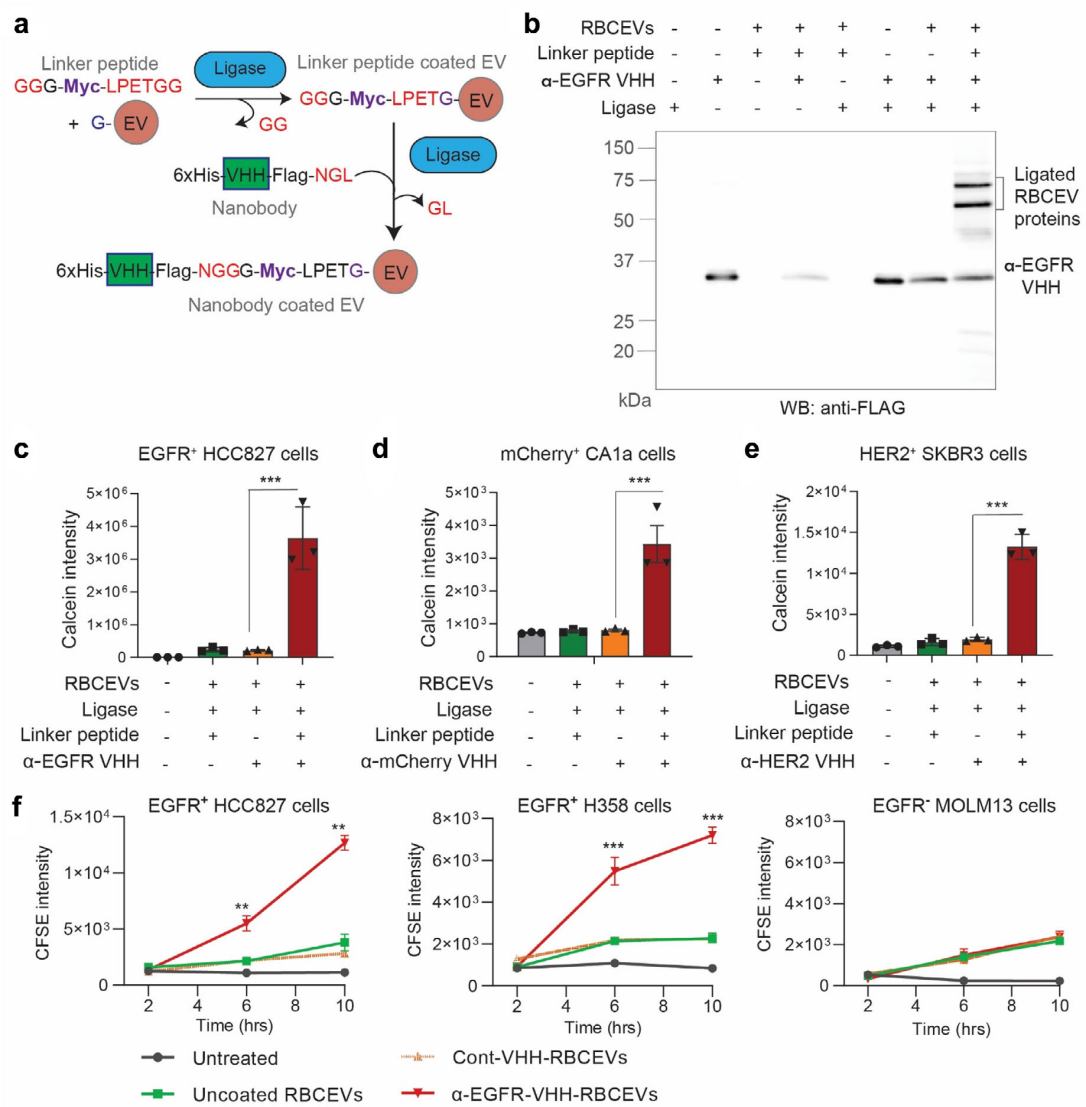
Nanobodies are conjugated to RBCEVs via a linker peptide, increasing the specific uptake of RBCEVs. **(a)** Two‐step conjugation of RBCEVs with nanobodies: EVs were first ligated with a linker peptide which was then ligated to a VHH nanobody. **(b)** Western blot analysis of α‐EGFR VHH (using α‐FLAG‐tag antibody), with or without conjugation to RBCEVs, after SDS‐PAGE separation. **(c)** Uptake of Calcein‐labelled α‐EGFR‐VHH‐ligated RBCEVs by EGFR^+^ lung cancer HCC827 cells. **(d)** Uptake of Calcein‐labelled α‐mCherry‐VHH‐ligated RBCEVs by mCherry‐expressing breast cancer CA1a cells. **(e)** Uptake of Calcein‐labelled α‐HER2‐VHH‐ligated RBCEVs by HER2‐expressing breast cancer SKBR3 cells. **(f)** Uptake of CFSE‐labelled RBCEVs ligated with α‐EGFR or control (α‐mCherry) VHH after 2–10 h of incubation with EGFR‐positive HCC827 or H358 cells versus EGFR‐negative MOLM13 cells. Graphs in (c) – (f) present the mean intensity of Calcein AM or CFSE ± SEM (n = 3 donors), analyzed using FACS. Student's one‐tailed *t*‐test: **P < 0.01, ***P < 0.001

In addition to peptides, we sought to use nanobodies to guide the specific delivery of RBCEVs to target cell types, because nanobodies are single‐domain antibodies known for their high affinity, high specificity and ease of modification (Li et al., 2018). We purified an anti‐EGFR camelid biparatopic nanobody (also called variable homodimers, VHH) with an additional sequence containing a 6xHis tag, a FLAG tag, and a ligase binding site (Figure S1D) (Roovers et al., 2011). The purified α‐EGFR VHH nanobody was approximately 31 kDa (Figure S1D). To reduce the steric hindrance, which may prevent the ligase‐mediated conjugation of RBCEVs with the α‐EGFR VHH nanobody, we designed a linker peptide with three glycine residues at the N terminus and a sortase/ligase binding site at the C terminus to bridge the VHH and RBCEVs (Figure 6a). This design allows a sequential ligation of the linker peptide to RBCEVs first, and then to the VHH nanobodies (Figure 6a). The linker peptide was designed such that it does not circularize or form oligomers because OaAEP1 ligase can bind to the linker peptide's C‐terminal LPETGG motif and efficiently conjugate it to the N terminal of EV proteins, but not to its own GGG motif. RBCEVs were ligated to the α‐EGFR nanobody with or without an addition of the linker peptide and washed extensively using SEC and 4 rounds of centrifugation. We observed the free α‐EGFR nanobody as a ∼31 kDa band. After the two‐step VHH‐ligation to RBCEVs using the linker peptide, several protein bands, particularly two prominent bands at ∼60 and 75 kDa, were detected using an anti‐FLAG antibody (Figure 6b). No band, other than the unligated VHH band, was observed in the ligation reaction without the linker peptide, suggesting that the ligation of VHH nanobodies to RBCEVs required the linker peptide. The spectrum of α‐EGFR‐VHH‐ligated protein bands was consistent in RBCEVs from three independent blood donors (Figure S6A). By comparing the total intensity of these bands to a serial dilution of free α‐EGFR VHH, we estimated that each EV was ligated to ∼49 copies of α‐EGFR‐VHH on average (Figure S6A). FACS analysis of His tag on RBCEV‐bound beads suggested that a large fraction of RBCEVs were conjugated to α‐EGFR‐VHH via the linker peptide (Figure S6B). This observation was confirmed using FACS analysis of FLAG tag on single EVs, showing ∼30% of the ligated EVs with VHH (Figure S6C). Nanobody conjugation was less efficient than peptide conjugation, probably due to the larger size of nanobodies that limits their number on the surface of each EV.

### 2.6 Conjugation of EVs with nanobodies promotes their specific uptake by target cell types

Consistently, we observed a significant increase in RBCEV uptake by HCC827 cells only when RBCEVs were conjugated with α‐EGFR VHH via the linker peptide (Figure 6c). Although the nanobody conjugation to RBCEVs was less efficient than the peptide conjugation, the increase in RBCEV uptake by the α‐EGFR VHH conjugation was comparable to that by the ET peptide conjugation, probably due to the higher affinity of α‐EGFR VHH towards EGFR (Li et al., 2005; Li et al., 2018). Using the same method, we found that conjugation of RBCEVs with α‐mCherry VHH and with α‐HER2 VHH promoted RBCEV uptake by mCherry‐expressing CA1a cells and HER2‐expressing SKBR3 cells, respectively (Figure 6d‐e). α‐mCherry and α‐HER2 VHH were expressed in *E. coli*, purified using FPLC (Figure S1E‐F) and validated for their specific binding to CA1a‐mCherry and SKBR3 cells, respectively (Figure S7A‐D). Omission of the linker peptide in the VHH ligation reaction abrogated the increase in targeted RBCEV uptake (Figure 6c‐e). Hence, the two‐step ligation is a robust method to ligate RBCEVs with various nanobodies.

The α‐EGFR VHH exhibited a very high affinity to EGFR‐positive cells, including H358 and HCC827 cells, but not to EGFR‐negative MOLM13 cells (Figure S7E). Hence, RBCEVs conjugated with α‐EGFR VHH bound specifically to H358 and HCC827 cells but not to MOLM13 cells (Figure S8A‐C). This binding was detected using FACS analysis of GPA, a specific marker of RBCEVs, on the surface of the cells after 1 h of incubation with α‐EGFR conjugated RBCEVs at 4°C, suggesting that the binding did not require energy and RBCEVs were not internalized at this temperature (Figure S8A‐C). During an incubation at 37°C, α‐EGFR‐VHH‐ligated RBCEVs were taken up specifically by H358 and HCC827 cells, but not MOLM13 cells (Figure 6f). This trend of specific targeting of EGFR‐positive cells by α‐EGFR‐VHH‐RBCEVs held true over time (Figure 6f).

**FIGURE 7:**
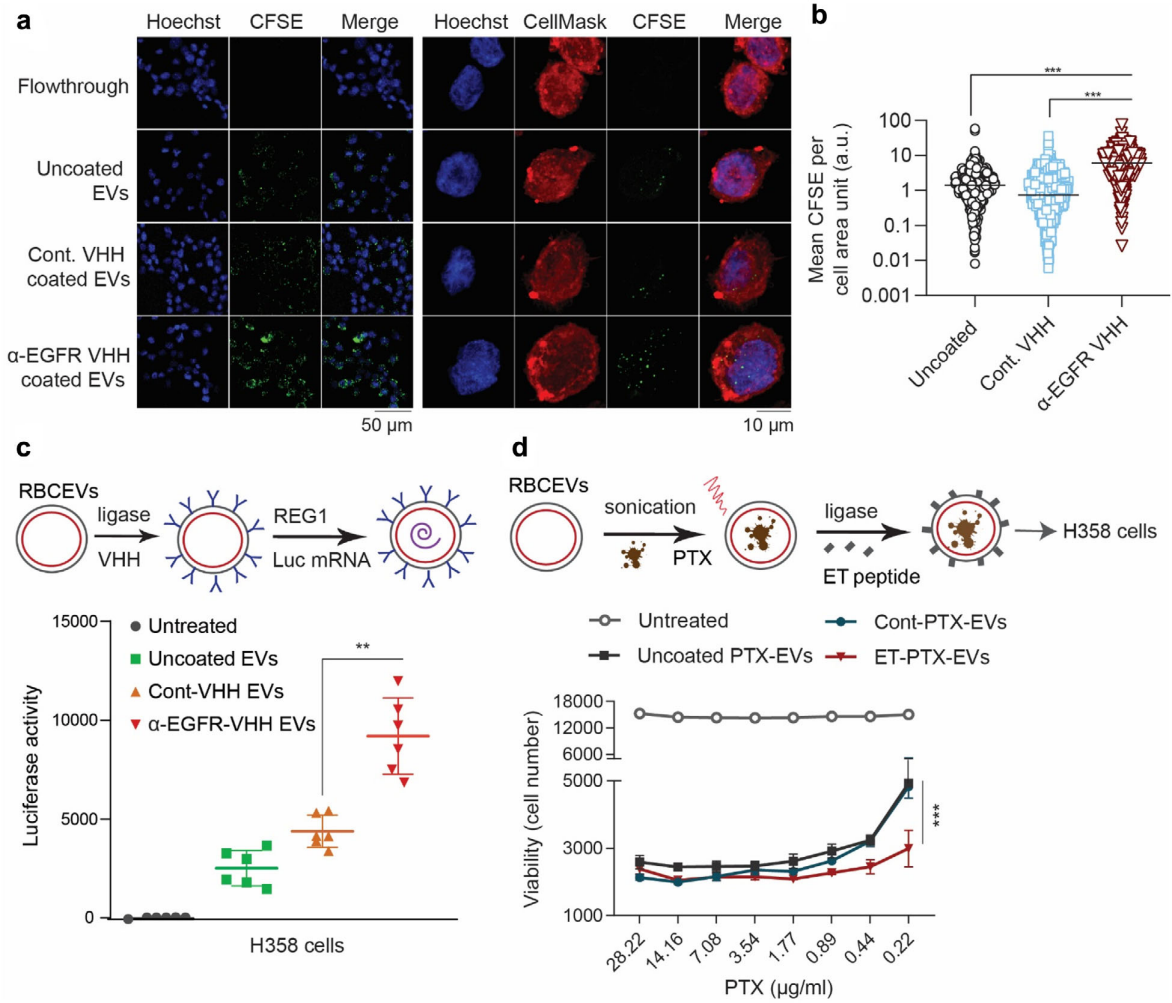
Conjugation of RBCEVs with targeting nanobodies and peptides facilitates cell‐specific delivery of therapeutic payloads. **(a)** Representative images of H358 cells that have taken up CFSE‐labelled RBCEVs, also stained with CellMask (red) and Hoechst (blue). **(b)** Mean CFSE signal per cell area unit was determined from the quantification of CFSE intensity in 1200‐1600 cells per condition. **(c)** Delivery of luciferase‐expressing (luc) mRNA using control (α‐mCherry‐VHH) or α‐EGFR‐VHH‐ligated RBCEVs, quantified based on luciferase activity in H358 cells after a 24‐h incubation with mRNA‐loaded RBCEVs (uncoated or ligated with VHH). Luciferase mRNA was loaded in RBCEVs using REG1 loading reagent. Graph presents luciferase signal ± SEM (n = 6 repeats). **(d)** Delivery of anti‐cancer drug paclitaxel (PTX) to lung cancer cells using ET‐peptide coated RBCEVs. PTX was loaded into RBCEVs using sonication. The loading capacity, percentage of PTX in RBCEVs (by weight) was determined using HPLC. Viability of H358 cells treated with different concentrations of PTX delivered by ET‐peptide‐ligated RBCEVs was calculated based on CCK8 assay readings ± SEM (n = 3 EV donors). Student's one‐tailed *t*‐test: **P < 0.01, ***P < 0.001

In addition, the increased uptake of α‐EGFR‐VHH‐ligated RBCEVs in H358 cells was also confirmed using immunofluorescence analysis and confocal microscopy (Figure 7a‐b). The punctate pattern of CFSE signals suggests that α‐EGFR‐VHH‐ligated RBCEVs were taken up mainly by endocytosis, consistent with our observation with the ET‐peptide‐coated RBCEVs.

### 2.7 Surface‐modified RBCEVs promote specific delivery of RNA and chemotherapeutic payloads

We have shown before that RBCEVs could be used to deliver ASOs, gRNAs or mRNAs to cancer cells (Usman et al., 2018). Here, we sought to test if the ligation reaction would affect RNA loading into EVs. We first conjugated RBCEVs with α‐EGFR or a control VHH (α‐mCherry‐VHH), washed RBCEVs twice using centrifugation, and subsequently loaded them with luciferase mRNA using REG1 (Figure 7c). H358 cells expressing EGFR, but not mCherry, were treated with these RBCEVs and the luciferase activity was compared after 24 h. The delivery of luciferase mRNA by α‐EGFR‐VHH‐RBCEVs resulted in 100% higher luciferase activity, compared to that by uncoated RBCEVs or α‐mCherry‐RBCEVs albeit all the RBCEV‐treated cells showed higher luciferase signals than untreated cells (Figure 7c). Thus, our data demonstrated that the conjugation of RBCEVs with α‐EGFR VHH enhanced the efficiency of mRNA delivery to EGFR‐positive cancer cells.

We also optimized a protocol for loading *PTX*, a generic chemotherapy drug commonly used for lung cancer treatments, into RBCEVs using sonication (Figure 7d). Drug‐loaded RBCEVs were washed thoroughly and ligated with the ET peptide. We observed that PTX was loaded into RBCEVs at a capacity of 5%–6% (in weight) and an efficiency of ∼25%, as determined by HPLC (Figure S8D). We treated H358 cells with a serial dilution of PTX‐loaded RBCEVs. Interestingly, we found a significant difference only at lower doses of PTX, suggesting that ET‐RBCEVs can enhance the efficacy of low dose PTX on EGFR‐positive cancer cells (Figure 7d).

### 2.8 EGFR‐targeting RBCEVs accumulate in xenografted EGFR‐positive lung cancer cells

**FIGURE 8:**
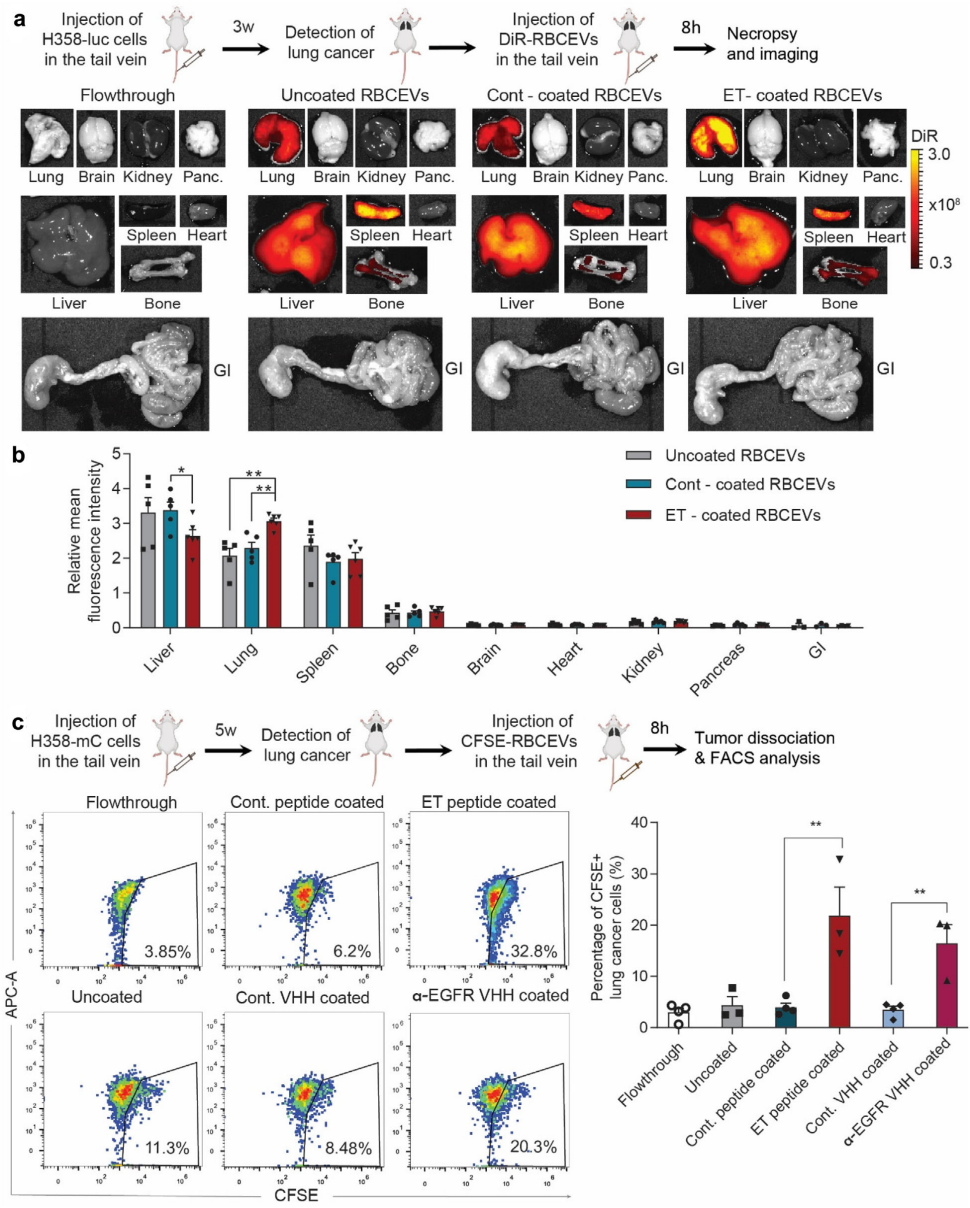
EGFR‐targeting RBCEVs accumulate in xenografted EGFR‐positive lung cancer cells. **(a)** Biodistribution of DiR‐labelled RBCEVs in NSG mice bearing EGFR^+^ H358 lung cancer. Shown are representative DiR fluorescent images of organs from lung‐cancer bearing mice preconditioned and injected with uncoated RBCEVs, control/ET‐RBCEVs or with the flowthrough of the RBCEV wash. **(b)** Average DiR intensity in each organ relative to the average mean intensity of all organs, subtracted by signals detected in flowthrough controls. Abbreviations: Panc, pancreas; GI, gastro‐intestinal tract. **(c)** *In vivo* uptake of CFSE‐labelled RBCEVs by mCherry‐positive H358 cancer cells, gated based on mCherry expression, in the lung of the mice that were treated with cont/ET‐peptide or cont/α‐EGFR‐VHH ligated RBCEVs, analyzed using FACS. Student's one‐tailed *t*‐test: *P < 0.05, **P < 0.01 (n = 3 to 5 mice)

To generate an *in vivo* model of lung cancer, we injected luciferase‐mCherry‐labelled H358 cells into the tail vein of NSG mice (Figure 8a). After 3 weeks, when tumour cells were stably engrafted and detected in the lungs, we treated the mice with DiR‐labelled RBCEVs and observed the biodistribution of RBCEVs by imaging. Since DiR is a fluorescent lipophilic dye, we found that it formed some micelles in PBS but the micelles and free DiR dye were eluted in separate SEC fractions (with low DiR signals in the DiR‐only sample), distinct from the fractions containing DiR‐labelled RBCEVs (fraction 7 to 10 with high DiR signals in the DiR‐EV sample) as shown in Figure S9A. We collected DiR‐labelled RBCEVs in fraction 7 to 9 for the *in vivo* biodistribution experiment because these fractions contained the highest concentration of RBCEVs (Figure S4C). The flowthrough of the last EV wash (the last round of centrifugation after the SEC) was used as a negative control to ensure that we could detect DiR fluorescent signals from RBCEVs above the background generated by any leftover unbound dyes. Bioluminescent cells were detected consistently in the lungs of NSG mice 3 weeks after the injection of H358‐luciferase cells, but no signal was detected in other organs, except occasionally in the tail due to the tail vein injection (Figure S9B). RBCEVs were conjugated with a control peptide or ET peptide using OaAEP1 ligase, then labelled with DiR fluorescent dye, washed extensively, and injected equally in the tail vein of tumour‐bearing mice. Eight hours after RBCEV injections, we observed uncoated RBCEVs in the spleen, liver, lung and bone (Figure 8a). Peptide‐coated RBCEVs showed similar pattern of uptake in the same organs. Remarkably, the accumulation of ET‐RBCEVs was significantly increased in the lung and reduced in the liver, compared to control‐peptide‐ligated RBCEVs (Figure 8a‐b). No significant change was observed in other organs (Figure 8a‐b). Conjugation of RBCEVs with ET peptide using Sortase A also led to an increased accumulation of EVs in the EGFR‐positive lung tumour (Figure S9C).

In order to analyze the specific uptake of RBCEVs by tumour cells *in vivo*, we labelled the peptide/VHH‐conjugated RBCEVs with CFSE and injected them into mice bearing mCherry‐H358 lung tumours (Figure 8c). After 8 h, we digested the lung tissue and analyzed the mCherry‐H358 tumour cells, using FACS. Interestingly, the conjugation of RBCEVs with ET peptide increased the percentage of CFSE‐positive tumour cells from 3%–6% in the controls to 20%–30% in the targeted ET‐RBCEV treatment (Figure 8c). Conjugation of RBCEVs with α‐EGFR VHH also increased the percentage of CFSE‐positive tumour cells to ∼20% in the targeted α‐EGFR‐RBCEV treatment (Figure 8c). These data suggest that both the ET peptide and the α‐EGFR VHH nanobody can drive RBCEVs specifically into EGFR‐positive lung tumour cells *in vivo*. Since the ET‐peptide‐conjugated RBCEVs performed better than α‐EGFR‐VHH‐conjugated RBCEVs in enhancing the specific uptake of the EVs, we further used ET‐peptide‐conjugated RBCEVs for drug delivery in the treatment of lung cancer shown below.

### 2.9 Delivery of paclitaxel (PTX) by EGFR‐targeting EVs enhances treatment efficacy in xenografted mice

**FIGURE 9:**
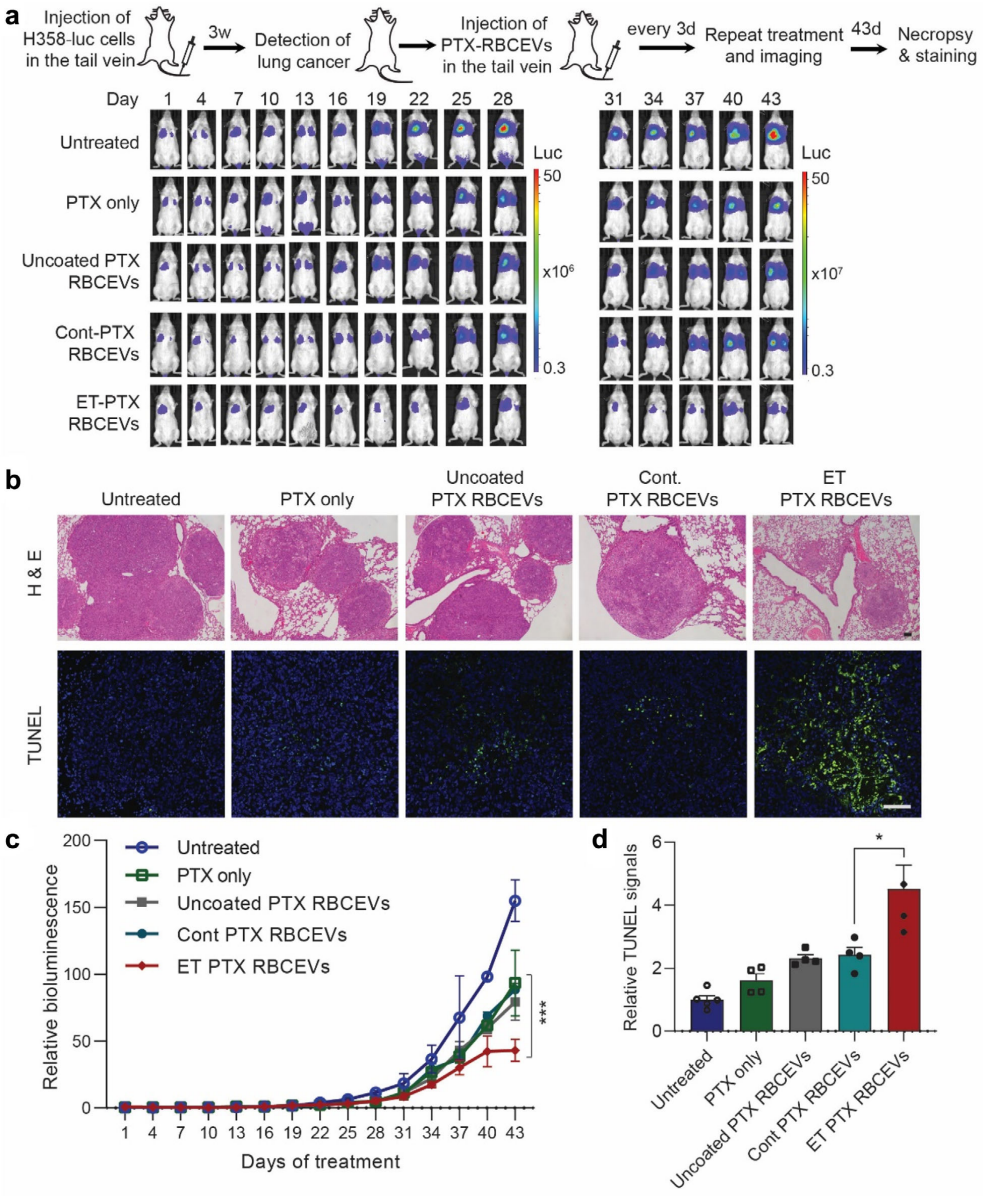
Delivery of PTX using EGFR‐targeting RBCEVs increases the treatment efficacy in an EGFR‐positive lung cancer mouse model. **(a)** Representative bioluminescent images of NSG mice with EGFR+ luciferase‐expressing H358 cancer cells in the lung during a course of systemic (i.v.) treatments with 1 mg/kg PTX only or the same dose of PTX loaded in cont/ET‐RBCEVs. Treatments were repeated every 3 days and images were taken 1 day after every treatment. Colours indicate bioluminescent signals (photon/s) in two scales (the images are divided into two groups, day 1–28 and day 31–43 from the treatment start date, to avoid saturated signals).**(b)** Representative images of H & E staining and TUNEL assay (green fluorescence) of lung sections from the lung cancer mice treated with PTX, with or without RBCEV‐mediated delivery. Nuclei were stained with DAPI (blue). Scale bar, 100 μm. **(c)** Average bioluminescent signals quantified in the lung area during the development of H358 lung tumours (photons/s), normalized by the signals at the start of the treatments, and presented as mean ± SEM. **(d)** Average fold change in TUNEL staining signals relative to the untreated control ± SEM. Two‐way ANOVA test (c) and Student's one‐tailed *t*‐test (d): *P < 0.05, ***P < 0.001 (n = 3 to 4 mice)

Subsequently, PTX‐loaded RBCEVs or an equivalent dose of free PTX (1 mg/kg) were injected into NSG mice bearing H358 lung cancer every 3 days (Figure 9a). Indeed, 1 mg/kg of PTX is considered a very low dose as the clinically‐equivalent dose of PTX is about 20 mg/kg in mice which causes many side effects (Wang et al., 2017). Bioluminescent imaging of the tumour revealed that ET‐RBCEVs significantly enhanced the tumour‐inhibitory effect of PTX, compared to the effect of free PTX or PTX loaded in uncoated and control‐peptide coated RBCEVs (Figure 9a, c). H & E staining and TUNEL staining of lung sections further confirmed that the treatment with ET‐RBCEVs containing PTX reduced the size of lung tumours while increasing apoptosis in these tumours compared to the treatment with free PTX or uncoated PTX‐loaded RBCEVs (Figure 9b, d). These data suggest that EGFR‐targeted RBCEVs can enhance the specific delivery of anti‐cancer drugs to targeted tumour cells *in vivo*.

## 3 DISCUSSION

In summary, we describe here a simple and robust protein ligase‐mediated method for conjugation of EVs with peptides and nanobodies to create a new generation of targeted therapies. Enzyme‐mediated surface functionalization of EVs has many advantages over existing EV modification methods, including: (1) no requirements for viral transduction and cell proliferation, thus reducing the cost and time for EV production; (2) no risk of transformation by viral insertion and DNA transfer; (3) stability of covalent bonds with peptides and nanobodies; (4) high‐purity peptides, enzymes and nanobodies can be produced in large scale at low cost; (5) ligase‐catalyzed reactions are reproducible and controllable with predictable rates and products; (6) ligases do not alter the physiochemical properties of EVs and the small amounts used can be removed easily by washing, hence the conjugation has no unwanted side effects.

We have shown that surface functionalization of EVs with target‐specific peptides and nanobodies can enhance the delivery of therapeutic molecules to cancer cells expressing the corresponding ligands, thereby increasing drug efficacy and decreasing side effects. We further demonstrated the versatility of this approach, both in terms of the variability of target ligands and the nature of the therapeutic molecules. Moreover, we showed that this targeting platform maintains functionality in *in vivo* settings. This indicates the potential for a versatile platform capable of targeting multiple ligands using EVs loaded with different types of therapeutic molecules for the treatment of a range of diseases.

We also demonstrated that modifying the surface of EVs with self‐peptide, which binds to SIRP alpha, can decrease phagocytosis, thereby increasing the bioavailability of EVs in circulation. This approach may also be applied to enhance the effects of EVs that have been proven to possess endogenous therapeutic properties, such as EVs from mesenchymal stem cells for cardiovascular diseases or EVs from dendritic cells for vaccination (Dang et al., 2020). Based on this study, we can surmise that conjugating antigens or biomarker‐binding (poly) peptides to EVs may facilitate future efforts in therapeutics and diagnostics.

Despite the versatility of this approach, there are certain limitations, presented primarily in the form of the targeting moieties used. Nanobody and peptide libraries are not very extensive and nanobodies with high affinity and specificity are not available against many cellular targets. Generation of novel nanobodies involves immunization and screening of phage display libraries which is a time‐consuming and tedious process. A more promising alternative is to develop a method for the stable conjugation of monoclonal antibodies to the EV surface, as monoclonal antibodies are readily available against most known antigens. There is also the risk of potential immunogenicity that could be introduced through the presence of camelid‐specific amino‐acid sequences in the nanobody (Vincke et al., 2009). If nanobody conjugated EVs are to be used for therapeutic applications, the nanobodies would need to be humanized by replacing these residues.

## 4 MATERIALS AND METHODS

### 4.1 Purification and characterization of RBCEVs

Blood samples were provided by Red Cross from healthy donors with informed consents in Hong Kong. Briefly, 200–250 ml of O‐group blood was collected in single‐system blood bags (Macopharma, France). Blood sample processing was performed according to the guidelines laid out by the City University of Hong Kong, following approval of all related procedures. RBCs were separated from plasma using centrifugation (1000 × *g* for 8 min at 4˚C) and washed three times with PBS (1000 × *g* for 8 min at 4˚C). White blood cells were removed by using centrifugation and leukodepletion filters (Nigale, China). Isolated RBCs were collected in Nigale buffer (0.2 g/l citric acid, 1.5 g/l sodium citrate, 7.93 g/l glucose, 0.94 g/l sodium dihydrogen phosphate, 0.14 g/l adenine, 4.97 g/l sodium chloride, 14.57 g/l mannitol) and diluted 3 times in PBS containing 0.1 mg/ml calcium chloride and incubated overnight with 10 μM calcium ionophore (Sigma, USA) at 37°C with 5% CO_2_. RBCEVs were purified using a similar procedure as described in our previous study (Usman et al., 2018), but with ultracentrifugation at 50,000 × *g*. Purified RBCEVs were stored in PBS containing 4% trehalose at ‐80˚C. The size distribution and concentration of RBCEVs were quantified utilizing a NanoSight NS300 Tracking Analysis NS300 nanoparticle tracking system (Malvern, UK) or a ZetaView Particle Tracking Analysis instrument (Particle Metrix, Germany). The haemoglobin contents of RBCEVs were measured using a haemoglobin quantification assay (Abcam, UK), comparing to a serial dilution of purified haemoglobin protein. Because haemoglobin is the major constituent of RBCEVs, RBCEV quantity is indicated by haemoglobin quantity throughout this study.

For transmission electron microscopy analysis, RBCEVs were fixed with 2% paraformaldehyde and loaded on a glow‐discharged copper grid (200 mesh, coated with formvar carbon film). RBCEVs on the grid were incubated with biotinylated anti‐human CD235ab antibody (catalogue number 306618, Biolegend, USA) followed by streptavidin protein (10 nm Gold conjugation, Abcam, ab81369). Samples were washed with PBS. A total of 4% uranyl acetate was added for negative staining of RBCEVs. Images were acquired using a Tecnai 12 BioTWIN transmission electron microscope (FEI/ Philips, USA) operating at 100 kV.

### 4.2 Purification of leukaemia EVs from THP1 cells

THP1 cells were obtained from the American Type Culture Collection (ATCC, USA) and maintained in RPMI (Thermo Fisher Scientific, USA), containing 10% foetal bovine serum (Biosera, USA) and 1% penicillin/streptomycin (Thermo Fisher Scientific). To make EV‐free FBS, EVs were removed from FBS using ultracentrifugation at 110,000 × *g* for 18 h at 4˚C. THP1 cells were cultured at 10^6^ cells/ml in the above medium with EV‐free FBS and 0.2 μM calcium ionophore for 48 h. Culture supernatants were collected from 5 flasks of treated THP1 cells. Cells and debris were separated by differential centrifugation at 300 × *g* for 10 min, 400 *× g* for 15 min, 900 × *g* for 15 min at 4˚C. The supernatant was further passed through a 0.45 μm filter, placed on top of 2 ml frozen 60% sucrose, and enriched by ultracentrifugation with a SW32 rotor at 100,000 × g for 90 min at 4˚C. THP‐1 derived EVs were collected from the interface and diluted 1:1 in cold PBS, and layered above 2 ml frozen 60% sucrose cushion in a SW41 rotor and centrifuged at 100,000 × *g* for 12 h at 4˚C (Beckman Coulter, USA) with reduced braking speed. 500 μl EVs were collected from the interface and added to a qEV SEC column (Izon, New Zealand). A total of 500 μl eluate was collected in each fraction. The concentration of EVs and protein were measured in 30 fractions using a Nanosight analyzer and bicinchoninic acid (BCA) assay (Pierce BCA Protein Assay Kit, Thermo Fisher Scientific). For ligation, the EVs from fraction 7 to 11 were combined and concentrated using centrifugation at 15,000 × *g* for 20 min in an Amicon‐15 filter with 100 kDa cut‐off.

### 4.3 Peptide and nanobody design

Peptides listed in Table S1 were designed with non‐targeting sequences as negative controls or with an EGFR‐targeting sequence or ‘self’ sequence obtained from previous reports (Li et al., 2005; Normanno et al., 2006). A sortase (LPETG) or ligase (NGL) binding motif was added to the C termini and a biotin was added to the N termini of the peptides. Peptides were synthesized using 96/102 well automated peptide synthesizers and purified using HPLC (GL Biochem Ltd., Shanghai, China). The EGFR‐VHH sequence was obtained from Roovers *et al*. (Li et al., 2018) and cloned with a 6xHis tag, a FLAG tag, and a ligase‐binding site in this order: 6xHis‐GSG‐VHH‐GSG‐FLAG‐NGL. Similarly, the sequences of an α‐mCherry and a α‐HER2 nanobody were obtained from Fridy *et al*. (Fridy et al., 2014) and Farasat *et al*. (Farasat et al., 2017) with an additional sequence as described above for α‐EGFR VHH. The VHH‐coding DNA was synthesized and inserted into pET32(a+) plasmid, following a T7 promoter, by Guangzhou IGE Biotechnology Ltd (China).

### 4.4 Expression and purification of proteins

Competent BL21 (DE3) *E*. *coli* bacteria were transformed with pET30b‐7 M‐SrtA plasmid (Addgene 51140, USA), OaAEP1‐Cys247Ala plasmid (provided by Dr. Bin Wu, Nanyang Technology University) or with pET32(a+)‐VHH plasmid (cloned with specific VHH sequences). Protein expression was induced using Isopropyl β‐D‐1‐thiogalactopyranoside (IPTG 0.5 mM) in LB at 25°C for 16 h with shaking. The culture was collected and centrifuged at 6000 × *g* for 15 min at 4°C. The bacteria were resuspended in 50 ml binding buffer (500 mM NaCl, 25 mM Tris‐HCl, 1 mM phenylmethylsulfonyl fluoride sulfonyl fluoride (PMSF), 5% glycerol). Bacteria were lysed using a high‐pressure homogenizer (JnBio, China) at 1000 psi for 4–6 rounds. The cell lysate was centrifuged at 8000 rpm for 60 min at 4°C. The supernatant was collected and filtered through a 0.45 μm membrane (Millipore, USA). The proteins were purified using a NGC‐QUEST‐10 fast protein liquid chromatography (FPLC) system (BioRad, USA). Briefly, the sample was loaded into a 5‐ml‐Ni‐charged cartridge (BioRad) equilibrated with the binding buffer. The column was washed with 3% elution buffer (500 mM NaCl, 25 mM Tris‐HCl, 1 mM imidazole, 1 mM PMSF and 5% glycerol) and then eluted using a linear imidazole gradient from 40 to 500 mM. Fractions of 2 ml were collected. The proteins were concentrated using a 3‐kDa‐cutoff centrifugal filter (Millipore) and 4000 ×g centrifugation in a swinging‐bucket rotor and filtered through a 0.22 μm membrane. The proteins were further purified using a HiLoad 16/600 Superdex 200 pg size exclusion chromatography column (GE Healthcare, USA) with the FPLC system, in low ionic strength buffer (150 mM NaCl, 50 mM Tris‐HCl), at 0.5 ml/min. The target protein was collected at the appropriate UV280 peak and validated using gel electrophoresis with Coomassie blue staining. For OaAEP1 ligase activation, a buffer comprised of 1 mM EDTA and 0.5 mM Tris (2‐carboxyethyl) phosphine hydrochloride was added to the immature protein and the pH of the solution was adjusted to 3.7 with glacial acetic acid. The mixture was incubated for 10 days at 4°C. Activated proteins were concentrated by ultracentrifugation using a 3‐kDa‐cutoff concentrator and stored at ‐80°C.

### 4.5 Conjugation of EVs with peptides using Sortase A or OaAEP1 ligase

For sortagging, 20 μM Sortase A was mixed with 500 μM peptide in 1x Sortase buffer (50 mM Tris‐HCl, 12 mM CaCl_2_, pH 6.5, 150 mM NaCl), and kept for 30 min. Subsequently, 100 μg RBCEVs (amount based on haemoglobin contents, equivalent to ∼5 × 10^10^ EVs) were added into the Sortase‐A‐peptide mixture in a total volume of 40 μl. We incubated the reaction for 1 h at room temperature with gentle agitation (20 rpm) on an end‐over‐end shaker. For ligation, 100 μg RBCEVs (∼5 × 10^10^ particles) were incubated with 500 μM peptide or VHH and 0.26 mg/ml ligase in PBS buffer, pH 7, in a total volume of 40 μl, at room temperature for 3 h with gentle agitation (30 rpm) on an end‐over‐end shaker. For VHH ligation, linker‐peptide‐ligated RBCEVs were washed twice with PBS and incubated with 500 μM VHH and 0.26 mg/ml ligase at the same condition as for the peptide ligation. After sortagging or ligation reactions, RBCEVs were washed with PBS using qEV SEC columns. Fraction 7 to 9 (with pink‐red colour) were collected and washed again with PBS 3 times by centrifugation at 21,000 × *g* for 15 min at 4˚C.

After washing, some peptide/nanobody‐coated RBCEVs were incubated with 10 μM Calcein AM (Biolegend) for 20 min at room temperature or with 20 μM CFSE (Life Technologies, USA) for 1 h at 37°C or with 2 μM DiR (Thermo Fisher Scientific) for 15 min at room temperature. All the dyes, including Calcein AM, CFSE and DiR, were dissolved in DMSO (Sigma) as 500x or 1000x stock solutions. The labelled RBCEVs were washed once using centrifugation and loaded into a SEC column (Izon) and eluted with PBS to wash the unbound dye away. RBCEVs, collected from SEC fraction 7 to 9, were washed twice with PBS by centrifugation at 21,000 × *g* for 15 min at 4˚C.

### 4.6 Loading RNAs and drugs into RBCEVs

1 μg luciferase mRNA (TriLink, USA) was loaded into 50 μg VHH‐ligated RBCEVs using REG1 (Carmine Therapeutics, Singapore) according to the manufacturer's instructions. mRNA‐loaded RBCEVs were then washed 3 times with PBS by centrifugation at 21,000 × *g* for 30 min. For drug loading, 1 mg uncoated RBCEVs were incubated with 200 μg PTX (Sigma) in 1 ml PBS at 37 ˚C for 15 min. The mixture was sonicated using a Bioruptor (Biogenode) for 12 min at 4˚C then recovered at 37˚C for 1 h. The loaded RBCEVs were washed with PBS at 21,000 × *g* for 15 min, quantified using the haemoglobin assay and coated with peptides as described above. The coated RBCEVs were repurified using SEC as described. To measure the amount of PTX loaded into RBCEVs, an aliquot of loaded RBCEVs was centrifuged at 21,000 × *g* for 15 min. The pellet was dried at 75˚C and resuspended in acetonitrile and centrifuged at 21,000 × *g* for 10 min. The supernatant was filtered and analyzed using HPLC.

To measure the effect of PTX‐loaded RBCEVs on cell proliferation, a serial dilution of RBCEVs containing 0.22 to 28.22 μg/ml PTX were added to each well of H358 cells in a 96‐well plate (5000 cells/well) at 37°C. After 60 h of incubation, the medium was replaced with 100 μl fresh medium containing 10 μl CCK8 solution (Biosharp, China) and incubated for 2 h at 37°C. CCK8 absorbance was measured at 450 nm using a Synergy H1 microplate reader (BioTek, USA).

### 4.7 Western blot analysis

Conjugated EVs were incubated with RIPA buffer (Thermo Fisher Scientific) supplemented with protease inhibitors (Biotool, USA) for 5 min on ice. A total of 50–200 μg protein lysates were loaded in 10% polyacrylamide gels and proteins were transferred to a polyvinylidene difluoride membrane (Immobilon‐P, Millipore). 3‐color protein ladder PM5100 ExcelBand (SmoBio, Taiwan) was included at two sides of the samples in the gel. Membranes were blocked using 5% milk in Tris buffered saline containing 0.1% Tween‐20 (TBST) for 1 h followed by an incubation with primary antibodies overnight at 4°C: mouse anti‐FLAG (Sigma, Cat# F3165, dilution 1:500). The blot was washed 3 times with TBST then incubated with HRP‐conjugated anti‐mouse secondary antibody (Jackson ImmunoResearch, USA, dilution 1:10,000) for 1 h at room temperature. For biotinylated peptide detection, the blot was incubated directly with HRP‐conjugated streptavidin (Thermo Fisher Scientific, dilution 1:4000). The blot was imaged using a BioRad Chemidoc gel documentation system.

### 4.8 Biotin pulldown and mass spectrometry analysis

Equal quantities of unmodified RBCEVs or biotinylated TR5 peptide ligated RBCEVs were lysed in RIPA buffer on ice for 10 min. The lysate from each sample was incubated with streptavidin‐magnetic beads from the Pierce MS‐Compatible Magnetic IP Kit (Thermo Fisher Scientific) for 1 h to allow binding of biotinylated proteins to the beads. The resulting beads were washed 3 times using a DynaMag‐5 Magnet (Thermo Fisher Scientific) in wash buffer before being subjected to denaturation in Laemmli buffer (BioRad). The beads were subsequently boiled at 95°C for 5 min. For total EV mass spectrometry, 100 μg RBCEVs were lysed in RIPA buffer on ice for 10 min. The protein lysate was denatured in Laemmli buffer and boiled at 95°C for 5 min. Biotin pulldown samples or RBCEV protein extracts were separated on a 12% NuPAGE Bis‐Tris gel (Life Technologies) for 10 min (LFQ) at 170 V in 1× MOPS buffer (Thermo Fisher Scientific). The gel was fixed using the Colloidal Blue Staining Kit (Thermo Fisher Scientific). For in‐gel digestion, samples were washed in the destaining buffer (25 mM ammonium bicarbonate; 50% ethanol), reduced in 10 mM DTT for 1h at 56°C followed by alkylation with 55 mM iodoacetamide (Sigma) for 45 min in the dark. Tryptic digest was performed in 50 mM ammonium bicarbonate buffer with 2 μg trypsin (Promega) at 37°C overnight. Peptides were desalted on StageTips and analyzed by nanoflow liquid chromatography on an EASY‐nLC 1200 system coupled to a Q‐Exactive‐HF mass spectrometer (Thermo Fisher Scientific). Peptides were separated on a C18‐reversed phase PicoFrit column (25 cm long, 75 μm inner diameter; New Objective) packed in‐house with ReproSil‐Pur C18‐AQ 1.9 μm resin (Dr Maisch). The column was mounted on an Easy Flex Nano Source and temperature controlled by a column oven (Sonation) at 40°C. A 105‐min gradient (biotin pulldown) or a 215‐min gradient (RBCEV proteomes) from 2% to 40% acetonitrile in 0.5% formic acid at a flow of 225 nl/min. Spray voltage was set to 2.2 kV. The Q‐Exactive‐HF was operated with a TOP20 MS/MS spectra acquisition method per mass spectrometry full scan. Mass spectrometry scans were conducted with 60,000 at a maximum injection time of 20 ms and MS/MS scans with 15,000 resolution at a maximum injection time of 50 ms. The raw files were processed with MaxQuant version 1.5.2.8 with standard settings for LFQ samples and the match between runs option was activated for the biotin pulldown samples (Cox & Mann, 2008). The quantification was based on unique peptides only. Carbamidomethylation was set as fixed modification while methionine oxidation and protein N‐acetylation were considered as variable modifications. Search results were filtered with a false discovery rate of 0.01. Known contaminants, proteins groups only identified by site, and reverse hits of the MaxQuant results were removed and only proteins that were quantified by LFQ intensity were kept.

### 4.9 Treatment of cancer cells with peptide or nanobody‐coated EVs

Human breast cancer SKBR3 cells, human lung cancer H358 and HCC827 cells were obtained from the American Type Culture Collection (ATCC, USA). Human breast cancer MCF10CA1a (CA1a) were obtained from Karmanos Cancer Institute (Wayne State University, USA). Acute myeloid leukaemia MOLM13 cells were obtained from DSMZ Collection of Microorganisms and Cell Cultures (Braunschweig, Germany). mCherry expressing CA1a cells were generated by our group as described previously (Vu et al., 2019). All the solid cancer and leukaemia cells were maintained in DMEM or RPMI (Thermo Fisher Scientific), respectively, with 10% foetal bovine serum and 1% penicillin/streptomycin (Thermo Fisher Scientific, USA). For the EV binding assay, 100,000 cells were incubated with 10 μg RBCEVs in 500 μl growth medium on an end‐over‐end shaker for 1 h at 4°C. The cells were washed twice with PBS and incubated with 1 μl APC anti‐GPA antibody (Biolegend Cat# 306608) for 1 h at 4°C, washed 3 times with PBS and analyzed by FACS. To test the EV uptake, 200,000 cells were incubated with 5 μg Calcein AM or CFSE‐labelled RBCEVs in 500 μl growth medium per well in 24‐well plates for 2–10 h at 37 °C. In the peptide competing assay, H358 cells were preincubated with 120 μM control or ET peptide for 1 h at 37°C then with 5 μg Calcein AM‐labelled RBCEVs in 500 μl growth medium per well in 24‐well plates for 2 h at 37 °C. To identify the route of EV uptake, we added EIPA, Filipin, or Wortmannin (at indicated concentrations) to H358 cells 1 h before adding Calcein‐AM‐labelled RBCEVs and incubated for 2 h as described above.

### 4.10 Flow cytometry (FACS) analysis

Cells were washed 2x with PBS, resuspended in 100 μl FACS buffer (PBS with 0.5% foetal bovine serum). For surface protein analysis, the cells were incubated with 3 μl fluorescent‐conjugated antibody such as AF488‐α‐EGFR antibody (Biolegend Cat# 352908), APC‐α‐His antibody (Biolegend Cat# 362605), APC anti‐GPA antibody (Biolegend Cat# 306608), APC‐α‐HER2 antibody (Biolegend Cat# 324407) for 15 min on ice, in the dark, and washed twice with 1 ml FACS buffer. To quantify the peptide conjugation efficiency, 100 μg biotinylated‐peptide‐coated RBCEVs or uncoated RBCEVs (as a negative control) were incubated overnight with 2.5 μg latex beads (Thermo Fisher Scientific) at 4°C on a shaker, washed three times with PBS and resuspended in 100 μl FACS buffer containing 1 μl streptavidin labelled with Alexa Fluor 647 (AF647), incubated on ice for 15 min and washed twice with FACS buffer. FACS analysis of latex beads or cells in FACS buffer was performed using a CytoFLEX‐S cytometer (Beckman Coulter) and analyzed using Flowjo V10 (Flowjo, USA). The beads or cells were first gated by FSC‐A versus SSC‐A, excluding debris and dead cells (Figure S4A). Single cells were then gated by FSC‐width versus FSC‐height, excluding doublets and aggregates. The fluorescent‐positive population of beads or cells was subsequently gated by targeted fluorescent channels, such as FITC for AF488, CFSE or Calcein AM, APC for AF647 and ECD for mCherry.

### 4.11 Single‐EV flow cytometry

Single‐EV flow cytometry was carried out using a CytoFLEX LX flow cytometer (Beckman Coulter). RBCEVs were distinguished from background noise via side scatter detected using the 405 nm laser (violet side scatter or VSSC) which allowed for greater sensitivity (Figure S3B). 100 nm and 200 nm control PCS mixed kit latex beads (Beckman Coulter) were used as a reference for size. Subsequently, RBCEVs were diluted in 0.2 μm‐filtered PBS and analyzed. Data was acquired using the following settings: FSC 138, SSC 180, FITC 3000, VSSC 800 with the threshold of the trigger signal (VSSC) set manually to 4000. The sample line was cleaned with sterile distilled water for 2 min at maximum flow rate (240 μl/min) before starting the experiment and between running each sample. Samples were analyzed at a slow flow rate (10 μl/min as measured by the machine), and analyzed only when the flow rate returned to the baseline level recorded at the start of the experiment to ensure that any remnants of the previous sample were not present and to maintain a constant level of background noise throughout the experiment. Staining of EVs was carried out at 4°C for 3 h, followed by 1 washing step using sterile filtered PBS to remove unbound antibody. Biotinylated peptide ligated EVs were detected by sequentially incubating ligated EVs with an excess of streptavidin (Abcam) followed by extensive washing and incubation with a biotinylated mouse isotype control monoclonal antibody (BioLegend). These EV‐TR5‐Streptavidin‐B‐antibody complexes were stained with an anti‐mouse‐AF488 conjugated secondary antibody (Jackson ImmunoResearch) at a concentration of 0.5 μg/ml. For staining of α‐EGFR VHH coated RBCEVs, 5 × 10^9^ EVs were incubated with 3 μl of antibody and the final volume made up to 300 μl with PBS. Uncoated EVs were also stained for each condition as controls. For the reagent/antibody control, antibodies were incubated in PBS under the same conditions as EVs and briefly spun down once before being analyzed. The buffer‐only control (filtered PBS), reagent control and all RBCEV samples were recorded at the same flow cytometer acquisition settings stated above, maintaining constant gain, triggering threshold, and flow rate. Following staining, EVs were resuspended to achieve a concentration of 2.5 × 10^5^ EVs/μl before being subjected to 2‐fold serial dilution 6 times. These serially diluted EV samples were analyzed by flow cytometry to establish the dilution range between which there was a linear relationship between dilution factor and EV count per minute (Figure S3D). In addition, the fluorescence of stained EVs was also measured at each dilution to ensure that the fluorescence signal was maintained constant, and that swarming did not take place. We found that EV suspensions within the range of 3.9 × 10^3^ to 2.5 × 10^5^ EVs/μl allowed analysis of single EVs accurately. As such, all EV samples were diluted to a concentration of 3 × 10^4^ EVs/μl before being analyzed at a flow rate of 10 μl/min, which yielded an average event rate of ∼5000 events/second. The abort rate was monitored throughout the experiment and kept within 0%–2%.

### 4.12 Immunostaining and imaging of RBCEV uptake

H358 cells were seeded on 13‐mm coverslips (Citoglass, China) coated with Poly‐D‐Lysine at a density of 20,000 cells per well (Sigma). After 2 days, the cells were incubated with 20 μg CFSE‐labelled RBCEVs coated with peptides or nanobodies in 500 μl medium for 4 h at 37°C, 5% CO_2_. After that, the cells were washed with PBS twice and incubated with 400 μl CellMask Deep Red (Thermo Fisher Scientific) per well for 10 min at 37°C. The cells were washed with PBS once and incubated with 400 μl 4% paraformaldehyde for 20 min at room temperature. The cells were rinsed twice with PBS before being stained with Hoechst 33342 (Cell Signalling Technology). Coverslips were washed three times with PBS and once with water and mounted on slides with mounting media (Abcam). Specimens were imaged using an inverted Zeiss LSM710 confocal microscope with 20X (0.5NA) air objectives and 100X (1.4NA) oil objectives. Images were acquired and analyzed using Zeiss Zen software (2011). Four to five random areas were taken using 20 × 0.5NA objective and subjected to semi‐quantification using Image J 1.8.0v software (National Institute of Health). Cell areas were selected as regions of interest (ROIs) based on the dilated mask of Hoechst signals. CFSE signals were measured as mean pixel intensity of ROIs. Total measurement area covered 1200 to 1600 cells in each condition.

### 4.13 Generation of *in vivo* cancer models and treatment with RBCEVs

All experiments in mice were conducted according to our protocols approved by the Institutional Animal Care and Use Committee under National University of Singapore and the Animal Ethics Committee at City University of Hong Kong. Mice of similar ages were tagged and grouped randomly for control and test treatments. Experiments were performed in a blinded manner. Exclusion was applied to the mice that became pregnant during the experiment or accidentally died due to anaesthesia. Mice with unsuccessful injections were also excluded to avoid false‐negative results.

H358 cells were transduced with a lentiviral vector (pLV‐Fluc‐mCherry‐Puro) and selected with puromycin to create a stable mCherry‐luciferase expressing cell line. A total of 1 million H358‐luc cells were injected into the tail vein of NSG mice (6‐7 weeks old). The same inoculation was repeated after 2 days. After 3 weeks, bioluminescence in the lung was detected using IVIS Lumina II (Perkin Elmer, USA) after an i.p. injection of D‐luciferin. Mice with comparable bioluminescent signals were injected with 1 mg RBC membrane lysate (ghosts) or an equivalent amount of live RBCs via a retro‐orbital route 1 h before RBCEV treatment.

To analyze RBCEV biodistribution, preconditioned mice with 3 week‐old lung tumours were injected with 100 μg DiR‐labelled RBCEVs, which were ligated with a control or EGFR‐targeting peptide, in the tail vein. After 8 h, mice were sacrificed and DiR fluorescence was measured immediately in the organs using the IVIS. Similarly, to analyze the specific uptake of RBCEVs by tumour cells, mice with 5‐week‐old lung tumours were injected with 800 μg CFSE‐labelled RBCEVs that were ligated with a control or EGFR‐targeting peptide/VHH in the tail vein. After 8 h, the mice were perfused with PBS and the lung was excised. Lung cells were dissociated using a gentleMACS dissociator (Miltenyi, Germany) and incubated with collagenase IV (Life Technologies) in a shaker for 40 min at 37°C. The cells were passed through a 70 μm strainer and centrifuged at 1500 rpm for 5 min. RBCs were lysed using ACK lysis buffer (Thermo Fisher Scientific). The rest of the cells were washed with PBS and resuspended in FACS buffer for FACS analysis of mCherry and CFSE.

For drug treatment, preconditioned mice were treated i.v. with 1 mg/kg PTX alone or an equivalent dose of PTX in RBCEVs with or without ET‐peptide ligation every 3 days. The same amount of unloaded RBCEVs was used as a negative control. Bioluminescent signals were measured every 3 days using IVIS as described above. At the end point (43 days from the beginning of the treatment, when the untreated mice showed many signs of pain and distress), the lung was fixed in 10% buffered formalin (Thermo Fisher Scientific) at room temperature overnight.

### 4.14 Quantification of RBCEVs in the circulation

500 μg CFSE‐labelled peptide‐ligated RBCEVs were injected into the tail vein of NSG mice. After 5–15 min, 100 μl blood was collected from the eye. Blood cells were removed and 20 μl plasma was incubated with 5 μl biotinylated anti‐GPA antibody (Biolegend Cat# 306618) for 2 h at room temperature with gentle rotation. The mixture was then incubated with 20 μl Dynabeads MyOne streptavidin beads (Thermo Fisher Scientific) for 1 h at room temperature. The beads were washed 3 times and resuspended in 500 μl FACS buffer for analysis of CFSE.

### 4.15 Histopathology analysis and TUNEL assay

After the overnight fixation, lung samples were sequentially dehydrated in 70%, 95% and 100% alcohol at 37°C. Samples were cleared in three baths of xylene (Thermo Fisher Scientific) and impregnated in 3 baths of paraffin wax (Thermo Fisher Scientific) each for 1.5 h at 37°C and 62°C, respectively. Paraffin blocks were sectioned at 5 μm using a HM330 MICROM microtome. Sections were dried at 37°C and dewaxed in 2 baths of xylene, then immersed in 2 baths of absolute alcohol and 1 bath of 70% alcohol, for 10 min each. Samples were rehydrated in water, stained with Gill 3 haematoxylin (Cancer Diagnostics, USA) for 15 min. After washing with water, the sections were treated with 0.3% acid alcohol, washed and blued with 2% sodium bicarbonate. Sections were subsequently stained with 0.5% Eosin (Merck, USA) for 2 min. After washing with water, samples were dehydrated in 95% and absolute alcohol, cleared in xylene then mounted using a synthetic mountant (Shandon). Apoptosis was evaluated using a TUNEL BrightGreen Apoptosis Detection Kit (Vazyme, China) according to the manufacturer's protocol. After staining, images of TUNEL staining were acquired with a confocal fluorescence microscope.

### 4.16 Statistical analysis

Student's *t*‐tests (one tail) were computed using GraphPad Prism 8 to determine significant differences between treated samples and control. Two‐way ANOVA, also computed using GraphPad Prism 8, was applied to analyze the differences among multiple groups of treatments. We considered a *P*‐value < 0.05 to be significant. In all the graphs, data are presented as median or mean and standard error of the mean (SEM). For statistical analysis, we repeated each experiment three times using RBCEVs from three donors or using cells from three passages. Animal experiments were performed in groups of 3 to 5 mice. The minimum sample size was determined by G*Power analysis, set for one‐tail t‐test that compared the mean difference of two independent groups with the α‐ err prob = 0.05, effect size d = 5, and power = 0.95.

## CONFLICT OF INTEREST

Minh TN Le and Jiahai Shi are scientific co‐founders, and advisors of Carmine Therapeutics. Other authors declare no conflict of interest.

## FUNDING

This project is funded by the National University of Singapore (grant NUHSRO/2019/076/STARTUP/02), the Hong Kong Innovation and Technology Commission (grant ITS/201/18), the Hong Kong Health and Medical Research Fund (05160296 and 06172196), the National Natural Science Foundation of China (81770099, 81773246, 81972865), the Shenzhen Innovation and Technology Fund (grant JCYJ20180507181636165, JCYJ20170413115637100 and JCYJ20170412152916724), the Hong Kong Research Grants Council (21101218), the Hong Kong Children's Thalassemia Foundation (2018/01), the National Research Foundation Singapore and the Singapore Ministry of Education under its Research Centre of Excellence initiative.

## GEOLOCATION INFORMATION

This project was performed in Singapore, Hong Kong, China and Vietnam.

## Supporting information

Supporting information.

Supporting information.

Supporting information.

Supporting information.

Supporting information.

## Data Availability

The mass spectrometry data have been deposited to the ProteomeXchange Consortium via PRIDE partner repository with the dataset identifier PDX021926. All raw data are available upon request.
