# Supplementary material for: Covalent conjugation of extracellular vesicles with peptides and nanobodies for targeted therapeutic delivery
Source: J Extracell Vesicles. 2021 Feb 16;10(4):e12057. doi: 10.1002/jev2.12057 (PMC7886705; doi:10.1002/jev2.12057)
Supplement: Supplementary file 1 — Supporting information. [file JEV2-10-e12057-s001.pdf]

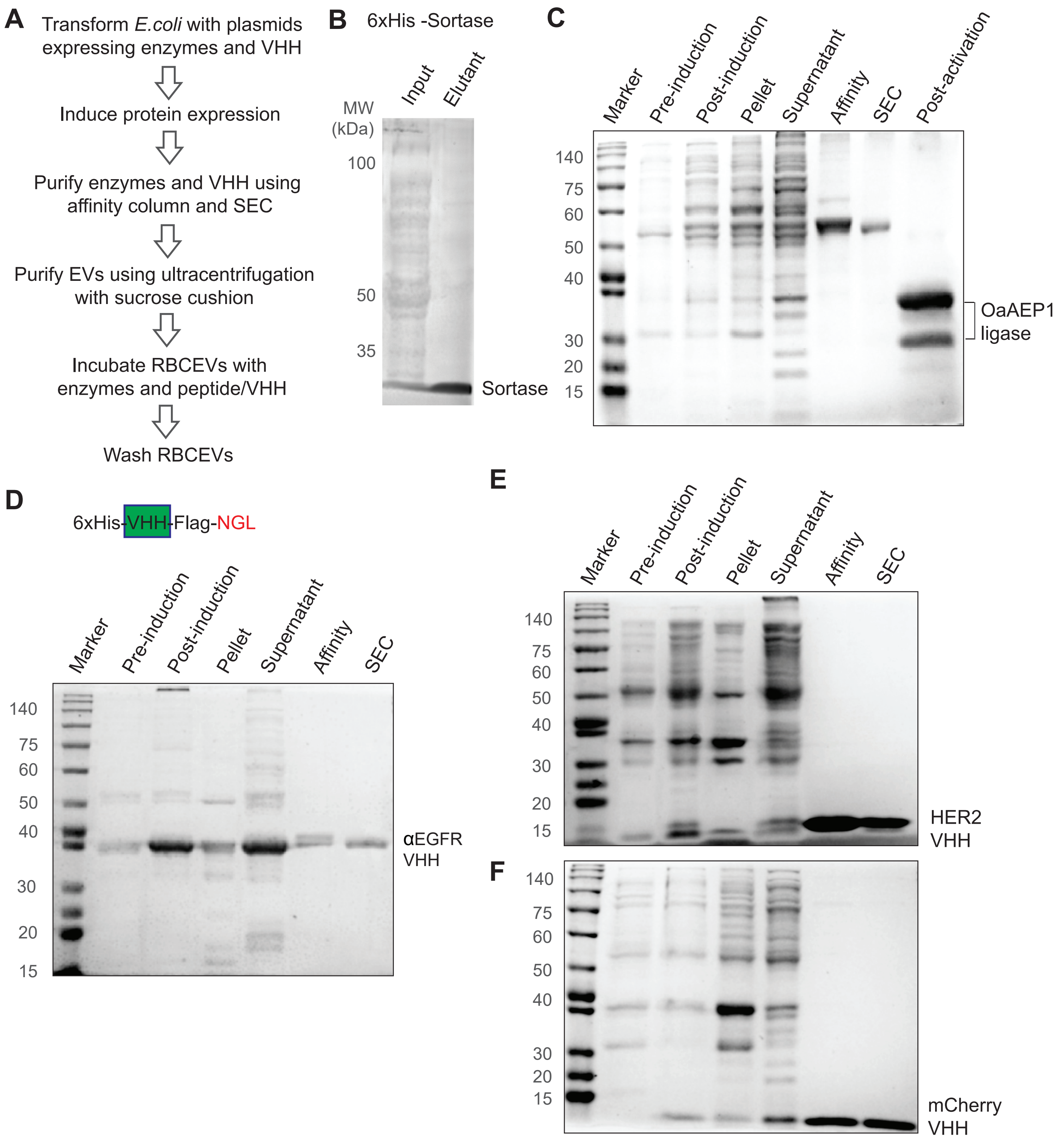

Figure S1| Purification of enzymes and nanobodies for EV conjugation. **(A)** Experimental workflow for protein purification and conjugation of RBCEVs. **(B)** SDS-PAGE analysis of proteins before (input) and after (eluent) FPLC purification of His-tagged sortase A (18kDa). **(C)** SDS-PAGE analysis of protein fractions obtained over the course of expression and FPLC purification (His-tag affinity and size exclusion chromatography, SEC) of OaAEP1-Cys247Ala ligase. **(D)** SDS-PAGE analysis of proteins obtained during FPLC purification of biparatopic α-EGFR VHH nanobody (~31 kDa) **(E)** SDS-PAGE analysis of proteins obtained during FPLC purification of α-HER2 VHH (~16 kDa) and **(F)** α-mCherry VHH (~15 kDa).

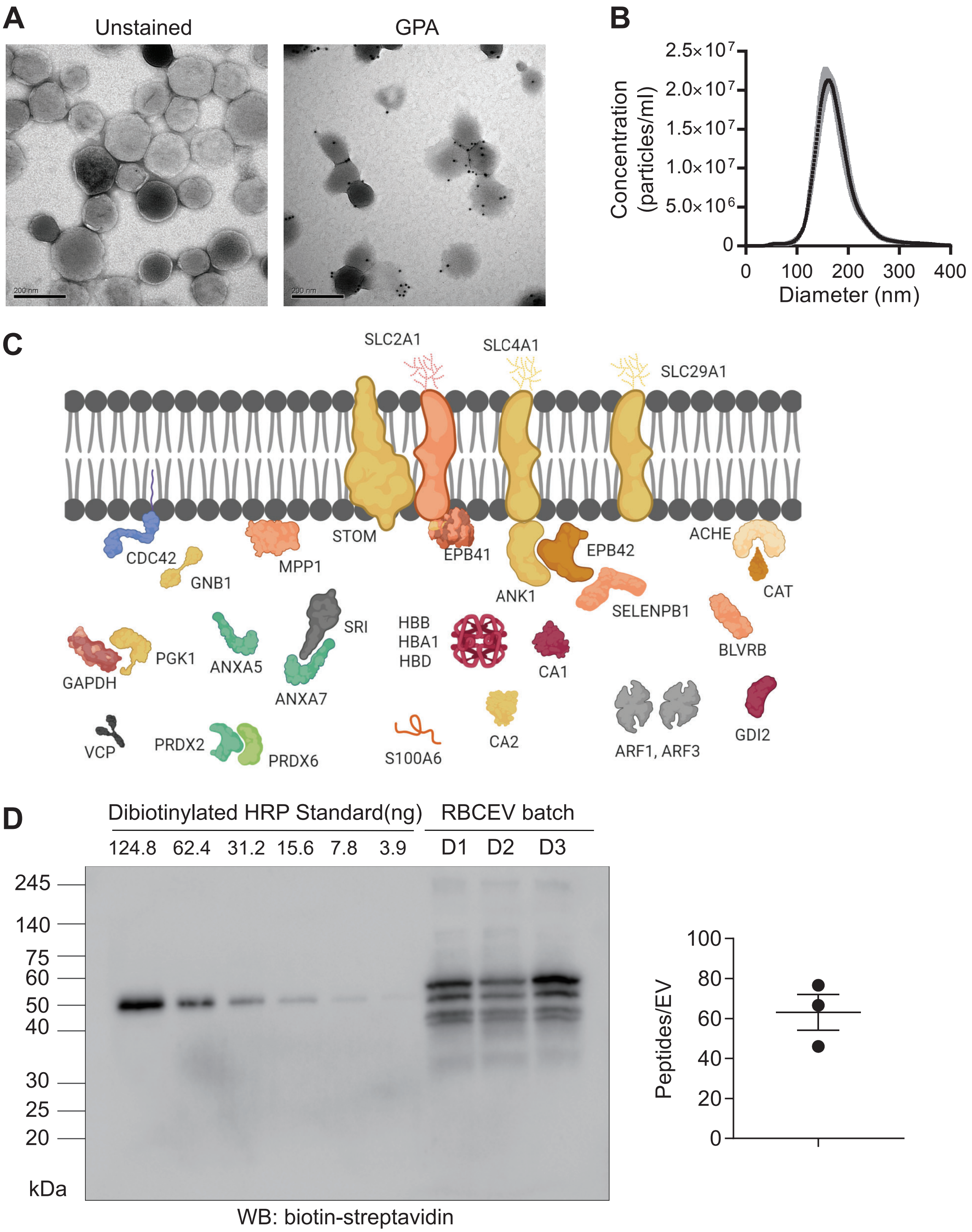

Figure S2| Characterization of unmodified and sortagged RBCEVs.
(A) Representative transmission electron microscopy images of RBCEVs unstained or stained with anti-GPA antibody. Scale bar, 200 nm. **(B)** Average concentration and size distribution of RBCEVs from 3 donors with the SEM in grey (100,000x dilution). **(C)** The 30 most abundant proteins in RBCEVs, identified using mass spectrometry. Interactions are depicted based on known interactions of the same proteins in RBCs. Image was created using biorender.com. **(D)** Western Blot analysis of RBCEVs from 3 different donors (D1-D3) conjugated with biotinylated GG8 peptide using Sortase A. Dibiotinylated HRP was used as a reference for quantification, and a particle analyzer was used to obtain the number of conjugated EVs loaded per well.

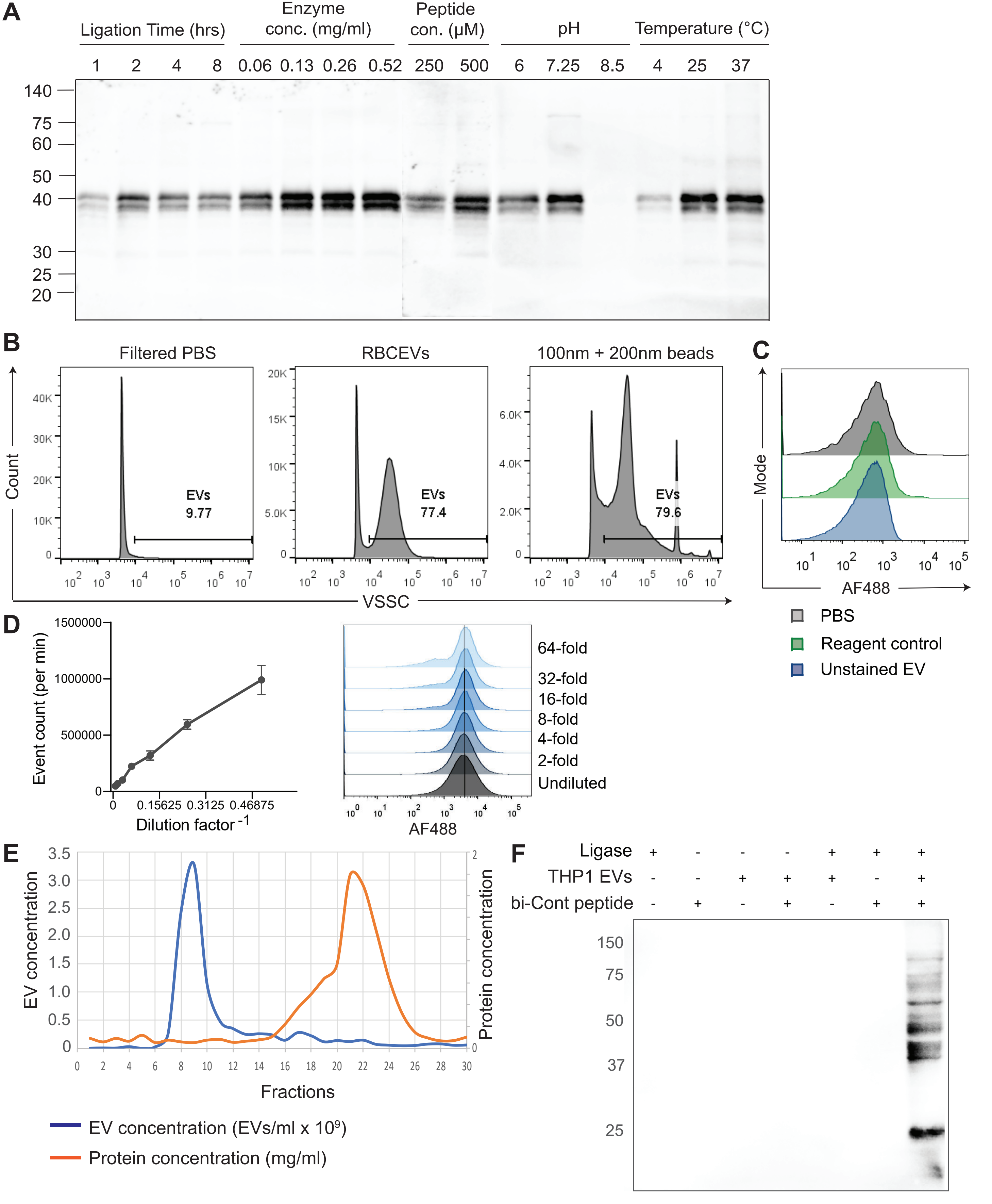

Figure S3| Optimization of EV-peptide conjugation using OaAEP1 ligase. (A) Western blot analysis of RBCEVs that were ligated with a biotinylated control peptide, under optimization for ligation time, enzyme concentration, peptide concentration, pH and temperature. Biotin was detected using immunoblotting with Streptavidin-HRP. **(B)** Gating strategy used to obtain a distinct population of RBCEVs from the background noise. 100 nm and 200 nm latex beads were tested as reference. **(C)** AF488 intensity of events in the EV gate of the reagent (antibody) control as compared to filtered PBS and unstained EVs. **(D)** Serial dilution of the RBCEVs, comparing event rate over a series of 7 dilutions and fluorescence intensity in the AF488 channel of stained TR5-ligated RBCEVs at the dilution range. **(E)** Size exclusion chromatography purification of EVs from THP1 cells, eluted in 30 fractions. EVs were detected using Nanosight particle analyser and protein concentration was measured using a BCA assay. **(F)** Western blot analysis of biotin associated with biotinylated control peptide ligated to EVs from THP1 cells, using OaAEP1 ligase.

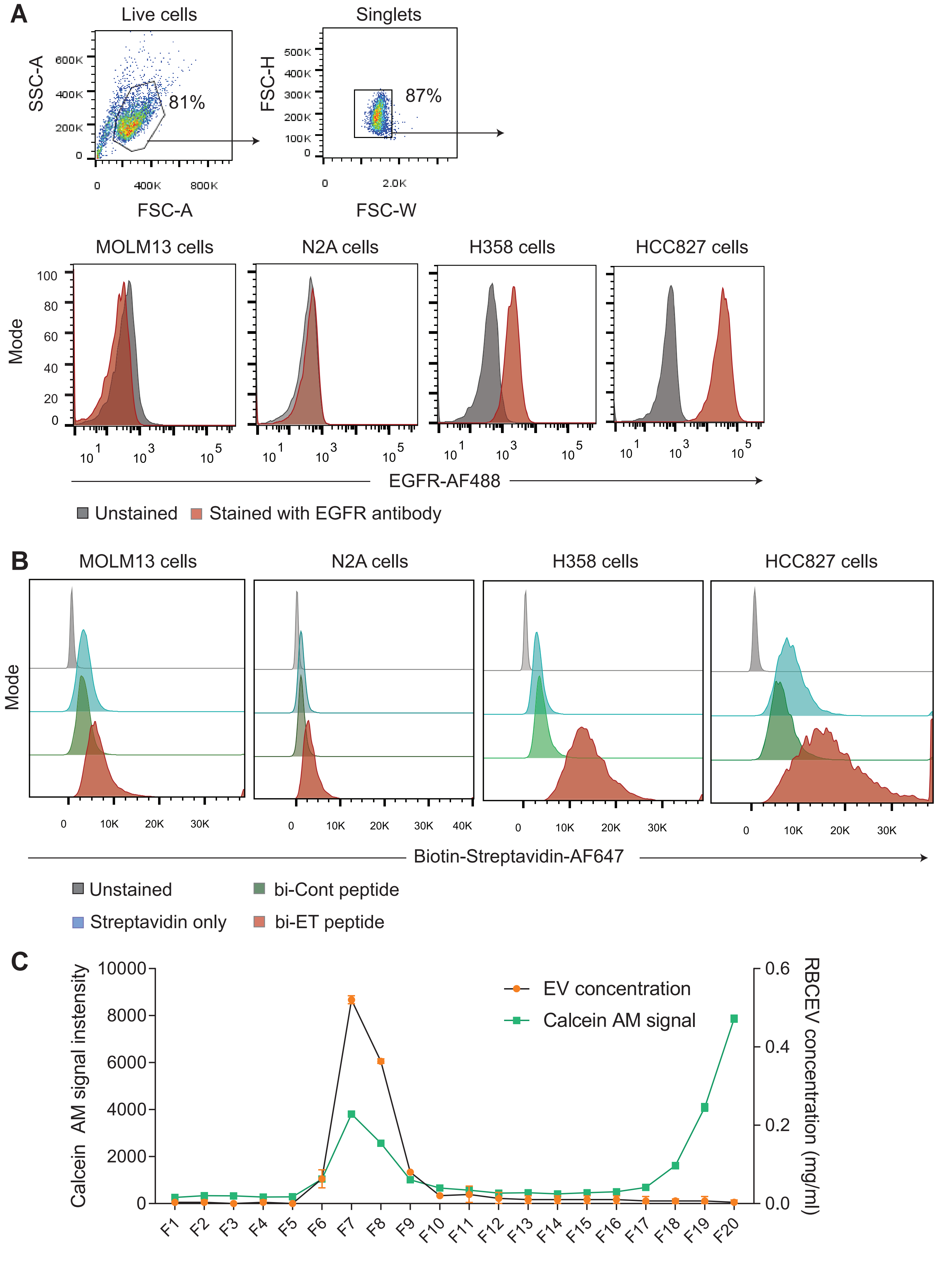

Figure S4. Specific binding of the EGFR-targeting peptide to EGFR-positive cells. (A) Expression of EGFR in human leukemia MOLM13 cells, mouse neuroblastoma N2A cells, human lung cancer H358 and HCC827 cells, analysed using FACS with a AF488 anti-EGFR antibody. **(B)** Binding of biotinylated control (Cont) or EGFR-targeting (ET) peptide to indicated cell lines, shown by a FACS analysis of biotin-bound AF647-streptavidin. **(C)** Separation of calcein AM dye from RBCEVs using SEC. EV concentration was determined using nanoparticle analyzer and calcein AM signal was measured using a spectrometer.

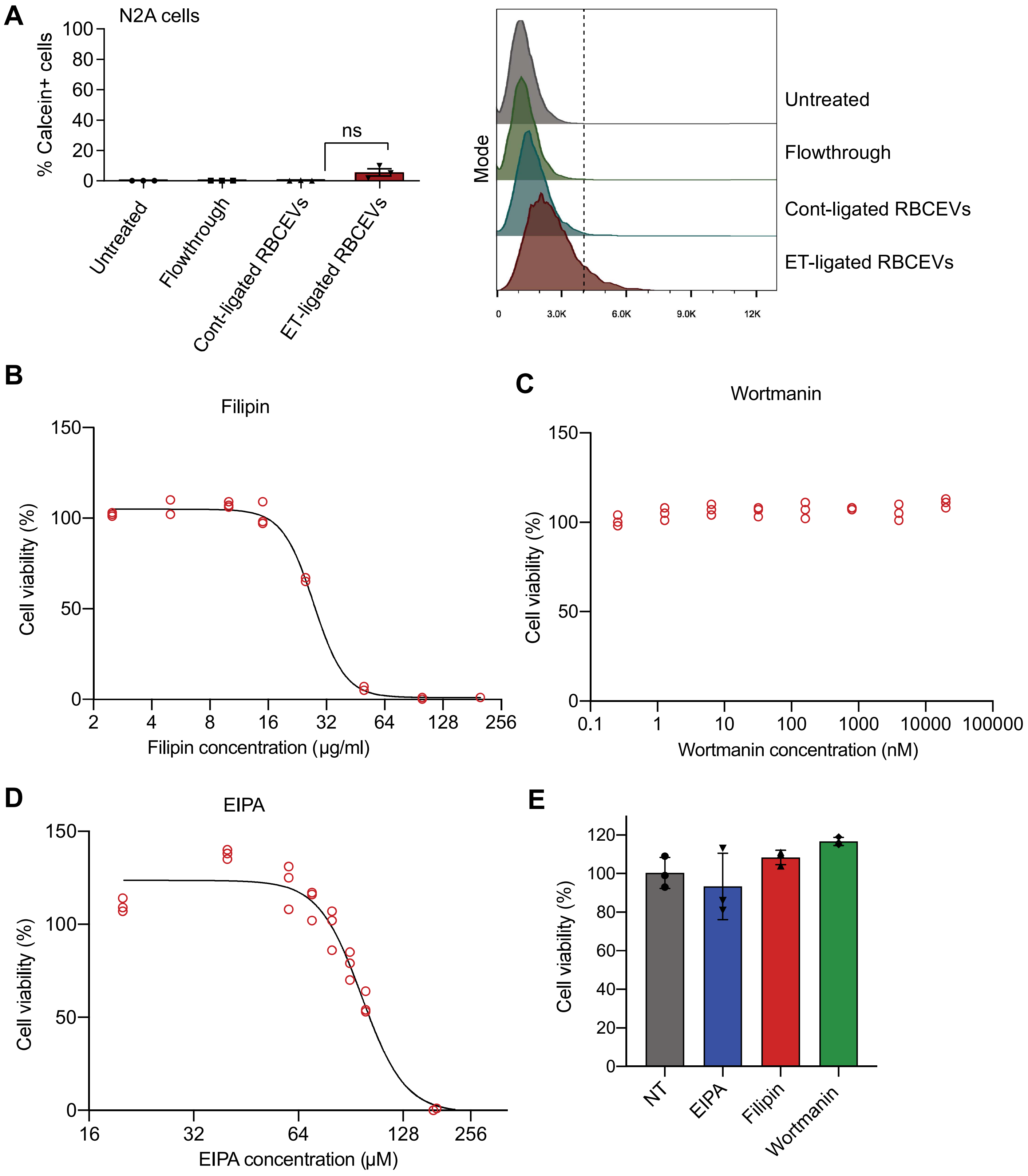

Figure S5| Uptake of RBCEVs by N2A cells and effects of endocytosis inhibitors on cell viability. (A) FACS analysis of calcein AM in N2A cells incubated with control or ET-peptide-ligated RBCEVs (n = 3 donors). **(B-D)** Effect of Filipin, EIPA and Wortmanin on the viability of H358 cells after 24 hours of treatment with indicated drugs at indicated doses. **(E)** Viability of H358 cells treated with 100 μM EIPA, 10 μg/mL Filipin, and 0.5 μM Wortmanin for 2 hours. Cell viability in (B)- (E) was determind using CCK8 assay.

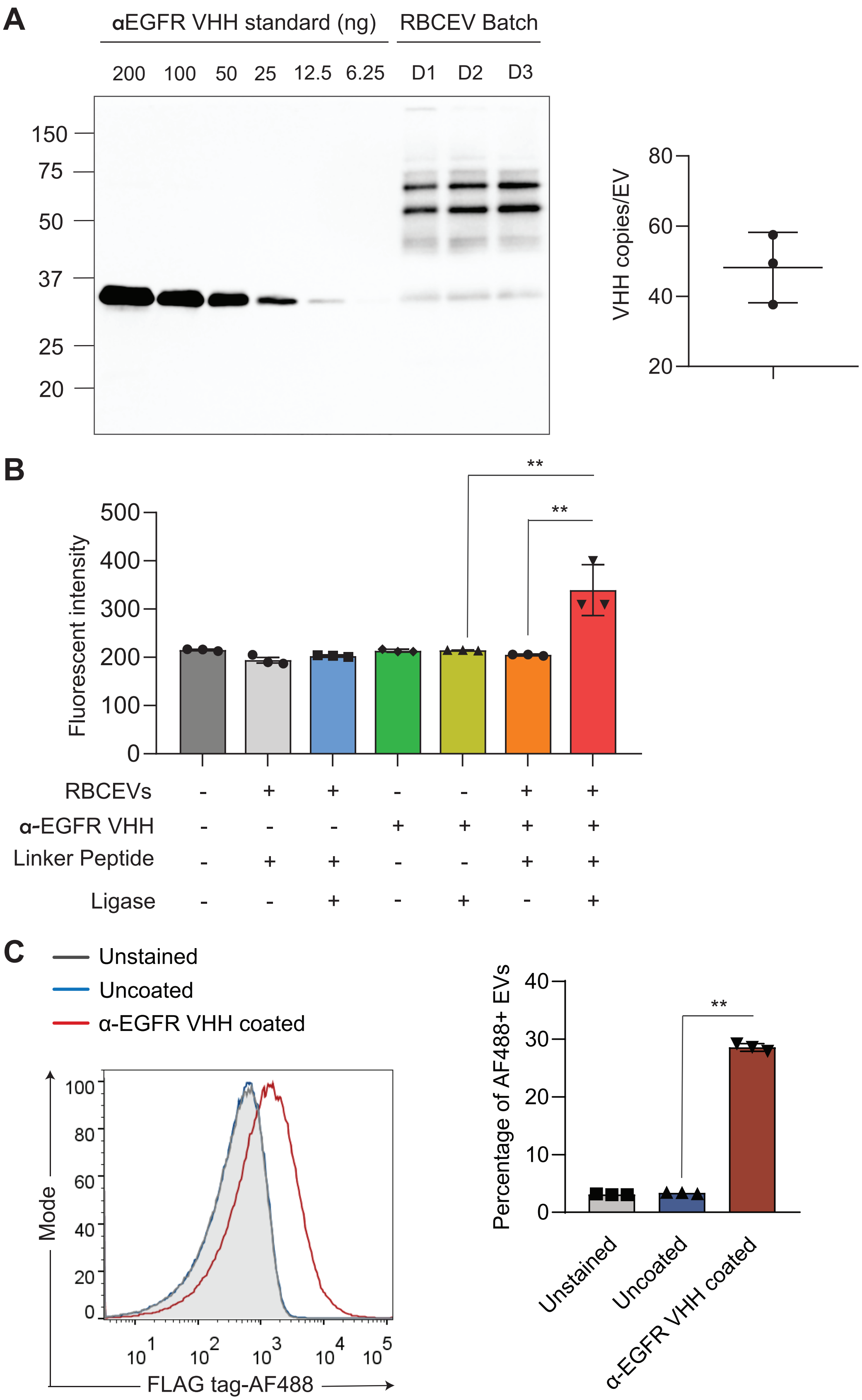

Figure S6| Efficiency of the nanobody-EV conjugation.
(A) Western Blot analysis of RBCEVs from 3 different donors (D1-D3) ligated with α-EGFR VHH via the linker peptide. Free α-EGFR VHH was used as a reference for quantification of nanobody copies per EV. **(B)** FACS analysis of His tag, an epitope tag of the α-EGFR VHH nanobody ligated to RBCEVs on anti-GPA streptavidin beads. **(C)** FACS analysis of FLAG tag, another epitope tag of α-EGFR VHH nanobody, on single RBCEVs Data are presented as mean intensity ± SEM (n = 3 EV donors). Student's t-test: **P<0.01.

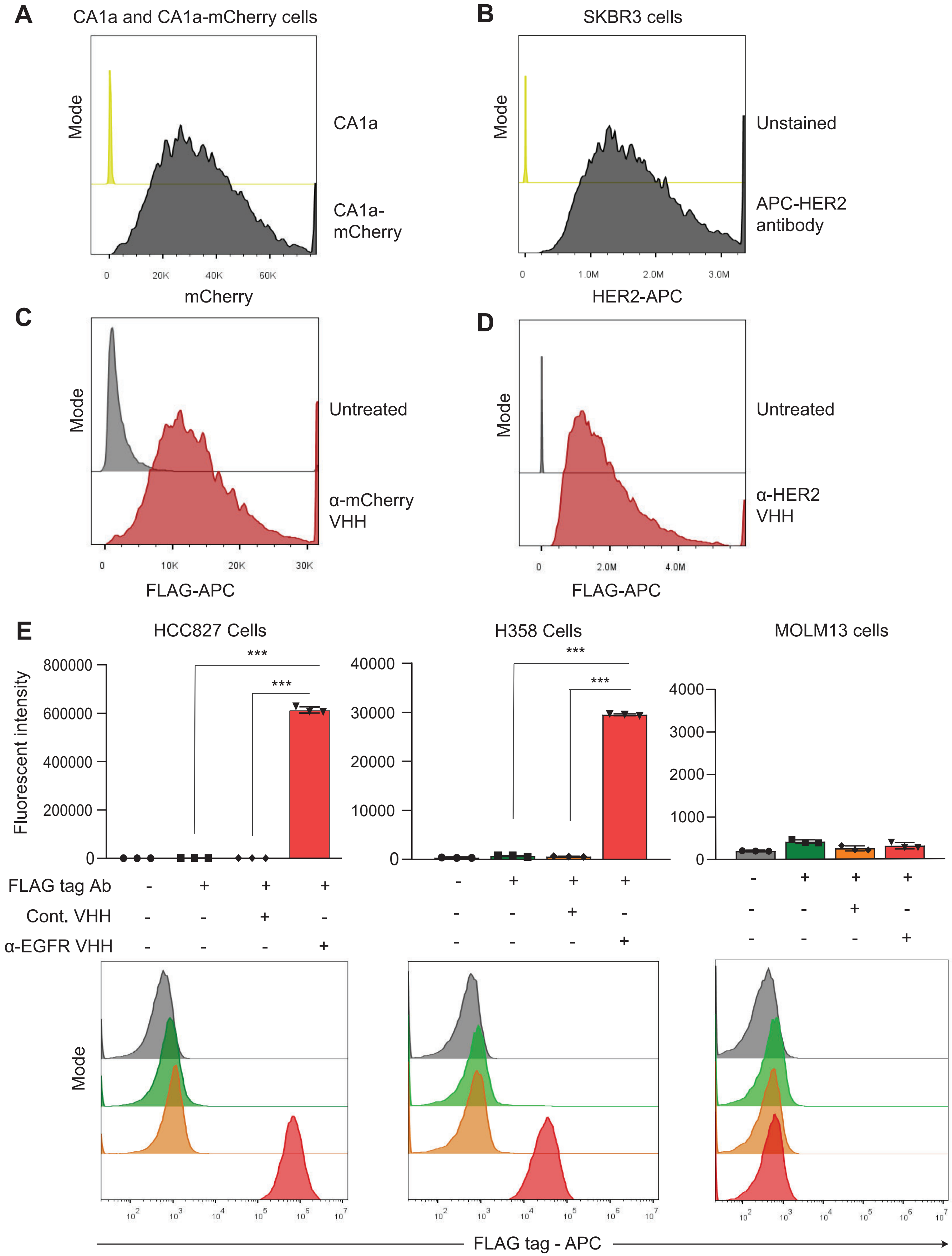

Figure S7| Specificity of nanobodies. (A) Expression of mCherry in parental and CA1a-mCherry cells. **(B)** Expression of HER2, detected using an α-HER2-APC monoclonal antibody in SKBR3 cells. **(C)** Binding of α-mCherry VHH to CA1a-mCherry cells detected by FACS analysis of FLAG tag on the cells after an incubation with α-mCherry VHH. **(D)** Binding of α-HER2 VHH to SKBR3 cells detected by FACS analysis of FLAG tag on the cells after an incubation with α-HER2 VHH. **(E)** FACS analysis of FLAG tag, an epitope tag on the α-EGFR VHH or control (α-mCherry) VHH nanobody that bound to EGFR-positive HCC827 and H358 cells versus EGFR-negative MOLM13 cells, using an APC α-FLAG tag antibody (Ab). Student's t-test: ***$P<0.001$ (n = 3 EV donors).

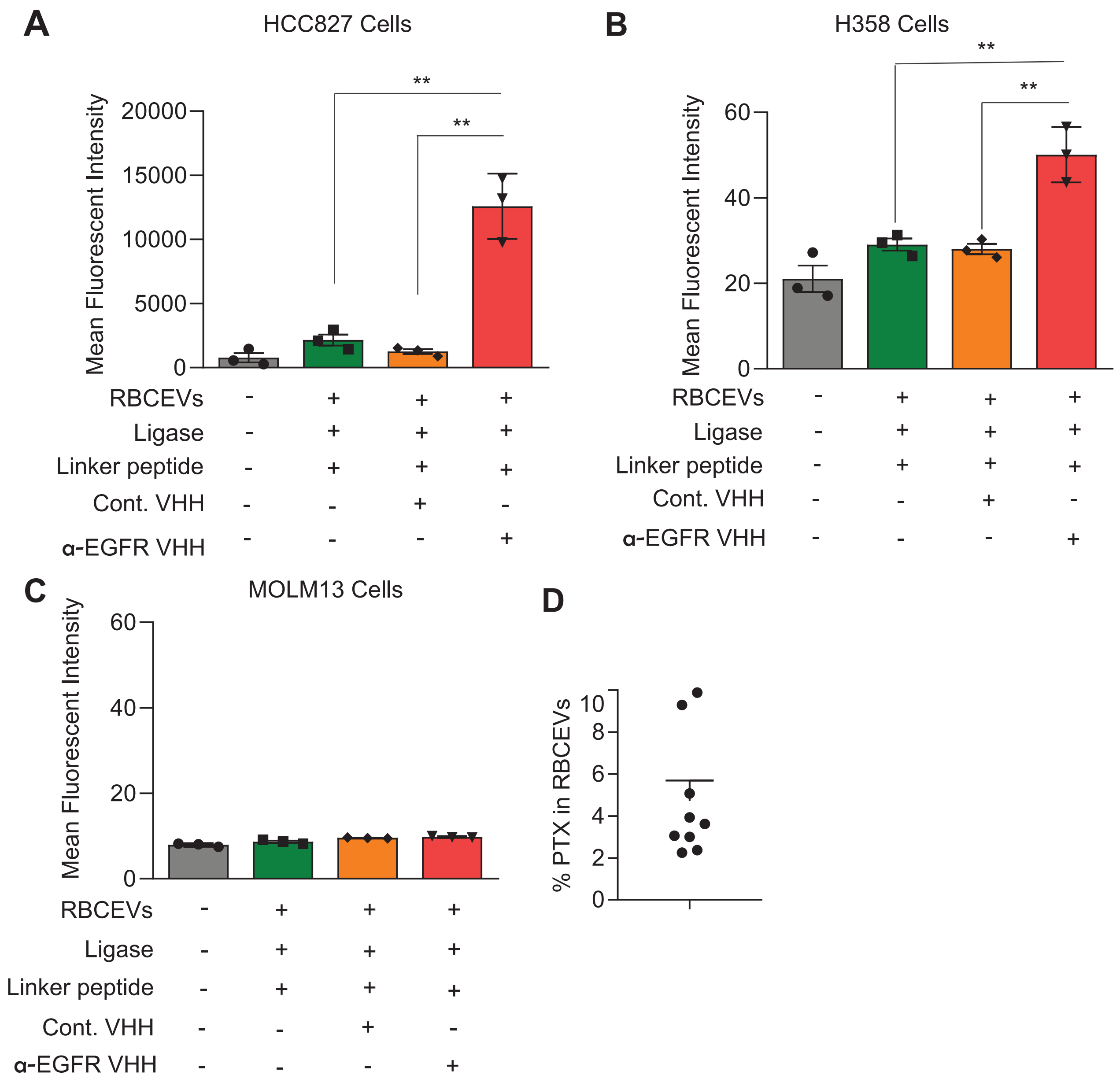

Figure S8| Specific binding of α-EGFR nanobody coated RBCEVs to EGFR-positive cells. (A-C) FACS analysis of GPA indicating the accumulation of RBCEVs on the surface of EGFR-positive HCC827 and H358 cells versus EGFR-negative MOLM13 cells after 1-hr-incubation of the cells with control (α-mCherry-VHH) or α-EGFR-VHH-ligated RBCEVs at 4°C. GPA was detected using an APC anti-GPA antibody, presented as mean intensity of APC ± SEM (n = 3 EV donors). Student's t-test: **P<0.01. **(D)** Loading capacity of RBCEVs with PTX (in weight).

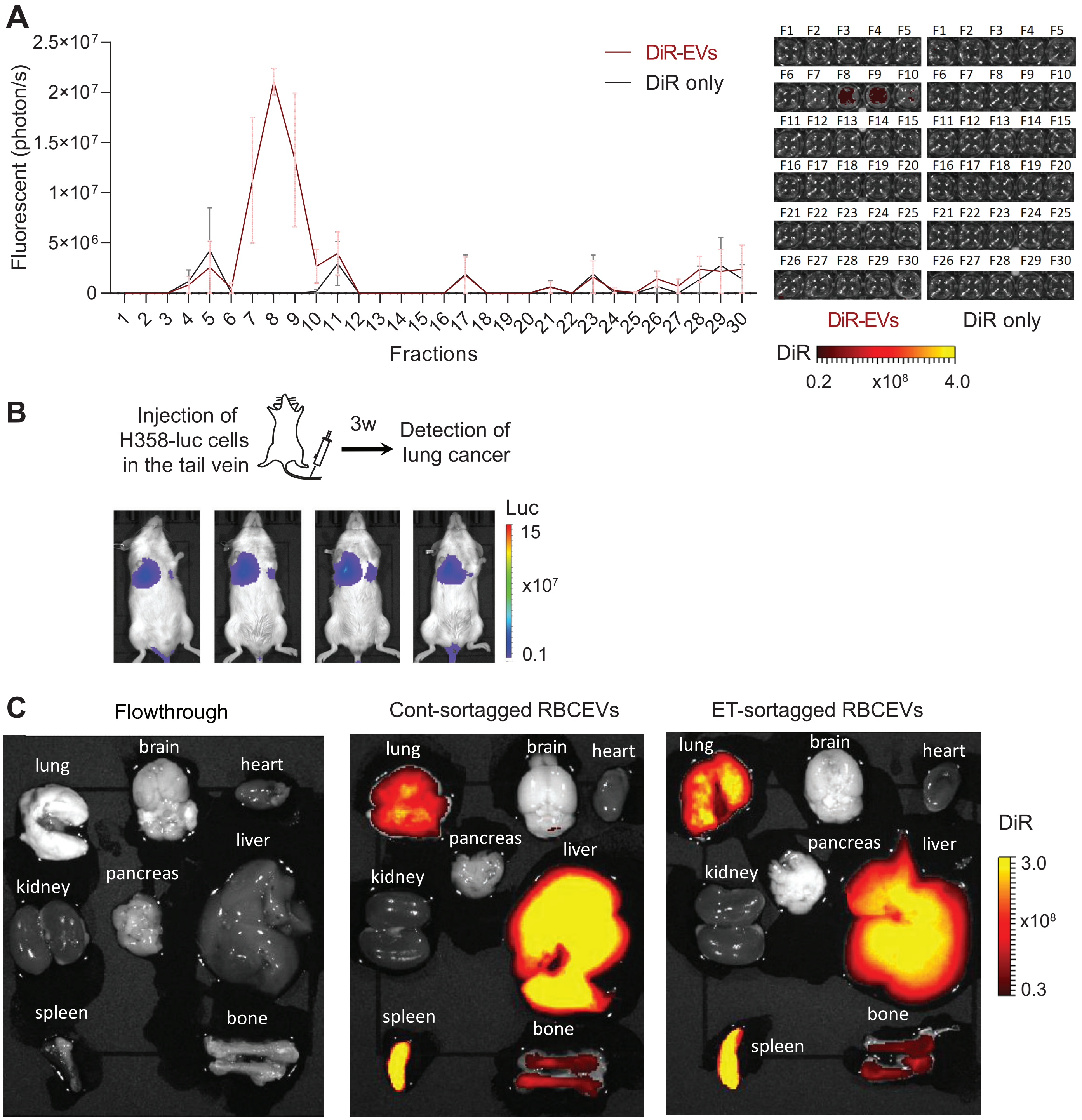

Figure S9| Biodistribution of EGFR-targeting RBCEVs in mice bearing EGFR-positive lung cancer. **(A)** Separation of DiR-labelled RBCEVs from DiR micelles (DiR only) in SEC fractions. Shown are the average DiR fluorescent signal in each fraction (n = 3) and representative images of the wells containing samples under IVIS. **(B)** Representative images of mice with lung cancer shown with bioluminescent signals in the lung 3 weeks after i.v. injection of H358-luciferase cells. **(C)** Representative DiR fluorescent images of organs from the mice injected with control/ET peptide-sortagged RBCEVs or with the flowthrough of the RBCEV wash.
